# Constrained peptides mimic a viral suppressor of RNA silencing

**DOI:** 10.1093/nar/gkab1149

**Published:** 2021-12-06

**Authors:** Arne Kuepper, Niall M McLoughlin, Saskia Neubacher, Alejandro Yeste-Vázquez, Estel Collado Camps, Chandran Nithin, Sunandan Mukherjee, Lucas Bethge, Janusz M Bujnicki, Roland Brock, Stefan Heinrichs, Tom N Grossmann

**Affiliations:** Chemical Genomics Centre of the Max Planck Society, Dortmund 44227, Germany; Department of Chemistry and Chemical Biology, Technical University Dortmund, Dortmund 44227, Germany; Department of Chemistry and Pharmaceutical Sciences, Vrije Universiteit Amsterdam, Amsterdam 1081 HZ, The Netherlands; Amsterdam Institute of Molecular and Life Sciences (AIMMS), Vrije Universiteit Amsterdam, Amsterdam 1081 HZ, The Netherlands; Department of Chemistry and Pharmaceutical Sciences, Vrije Universiteit Amsterdam, Amsterdam 1081 HZ, The Netherlands; Amsterdam Institute of Molecular and Life Sciences (AIMMS), Vrije Universiteit Amsterdam, Amsterdam 1081 HZ, The Netherlands; Department of Chemistry and Pharmaceutical Sciences, Vrije Universiteit Amsterdam, Amsterdam 1081 HZ, The Netherlands; Amsterdam Institute of Molecular and Life Sciences (AIMMS), Vrije Universiteit Amsterdam, Amsterdam 1081 HZ, The Netherlands; Department of Biochemistry, Radboud Institute for Molecular Life Sciences, Radboud University, Nijmegen Medical Center, Nijmegen 6525 GA, The Netherlands; Laboratory of Bioinformatics and Protein Engineering, International Institute of Molecular and Cell Biology in Warsaw, Warsaw 02-109, Poland; Laboratory of Bioinformatics and Protein Engineering, International Institute of Molecular and Cell Biology in Warsaw, Warsaw 02-109, Poland; Silence Therapeutics GmbH, Robert-Rössle-Str. 10, 13125 Berlin, Germany; Laboratory of Bioinformatics and Protein Engineering, International Institute of Molecular and Cell Biology in Warsaw, Warsaw 02-109, Poland; Department of Biochemistry, Radboud Institute for Molecular Life Sciences, Radboud University, Nijmegen Medical Center, Nijmegen 6525 GA, The Netherlands; Department of Medical Biochemistry, College of Medicine and Medical Sciences, Arabian Gulf University, Manama 293, Bahrain; University Hospital Essen, Institute for Transfusion Medicine, Essen 45147, Germany; Chemical Genomics Centre of the Max Planck Society, Dortmund 44227, Germany; Department of Chemistry and Chemical Biology, Technical University Dortmund, Dortmund 44227, Germany; Department of Chemistry and Pharmaceutical Sciences, Vrije Universiteit Amsterdam, Amsterdam 1081 HZ, The Netherlands; Amsterdam Institute of Molecular and Life Sciences (AIMMS), Vrije Universiteit Amsterdam, Amsterdam 1081 HZ, The Netherlands

## Abstract

The design of high-affinity, RNA-binding ligands has proven very challenging. This is due to the unique structural properties of RNA, often characterized by polar surfaces and high flexibility. In addition, the frequent lack of well-defined binding pockets complicates the development of small molecule binders. This has triggered the search for alternative scaffolds of intermediate size. Among these, peptide-derived molecules represent appealing entities as they can mimic structural features also present in RNA-binding proteins. However, the application of peptidic RNA-targeting ligands is hampered by a lack of design principles and their inherently low bio-stability. Here, the structure-based design of constrained α-helical peptides derived from the viral suppressor of RNA silencing, TAV2b, is described. We observe that the introduction of two inter-side chain crosslinks provides peptides with increased α-helicity and protease stability. One of these modified peptides (B3) shows high affinity for double-stranded RNA structures including a palindromic siRNA as well as microRNA-21 and its precursor pre-miR-21. Notably, B3 binding to pre-miR-21 inhibits Dicer processing in a biochemical assay. As a further characteristic this peptide also exhibits cellular entry. Our findings show that constrained peptides can efficiently mimic RNA-binding proteins rendering them potentially useful for the design of bioactive RNA-targeting ligands.

## INTRODUCTION

Viruses represent a universal infectious threat to all forms of life, from bacteria to fungi, plants and animals. In response, these organisms have evolved numerous mechanisms to fend off viral attack (1). One such defense mechanism in eukaryotes is RNA silencing, a central cellular process that triggers the degradation of viral double-stranded RNA (ds RNA) arising from replication intermediates or self-complementary viral genome sequences (2,3). In this process, Dicer-like proteins cleave long ds RNA into short fragments (19–23 bp long) known as small interfering RNA (siRNA). One of the two siRNA strands can then be incorporated into the RNA-induced silencing complex (RISC) which recognizes and cleaves complement viral messenger RNA (mRNA) (4). To suppress RNA silencing, viruses have evolved proteins which directly bind to siRNA structures thereby preventing RISC incorporation (5). Such viral suppressors of RNA silencing are diverse in structure and size, and belong to the large group of RNA-binding proteins (6–8). This group of proteins features a broad spectrum of functionalities beyond post-transcriptional regulation, and several efforts have been made to harness their scaffolds to design biomolecular tools that recognize specific RNA structures and sequences (9–13).

In contrast to the progress made in re-engineering RNA-binding proteins, the design of small molecular scaffolds that recognize RNA structures has proven very challenging (14–17). This is due to the unique structural features of RNA often involving dynamic and polar surfaces which tend to lack well-defined binding pockets. For this reason, alternative scaffolds of intermediate size have been exploited (18–20). Among those, peptide-derived molecules represent appealing entities as they can mimic structural features found in RNA-binding proteins. To date, peptide-based approaches have seen limited RNA-targeting applications. Examples have used the HIV–1 RNA-binding peptides Tat and Rev as starting points to apply different strategies to stabilize their bioactive conformation including lactam-bridge formation, head-to-tail cyclization, and thioether crosslinking (21–24). Moreover, the screening of modified peptide libraries has resulted in the discovery of novel RNA-binding ligands (25–27). However, the cellular uptake of such peptide-derived ligands has been a limiting factor.

For the development of inhibitors of protein-protein interactions, peptide-derived ligands have been widely used (28). In those cases, protein fragments have been stabilized in their bioactive conformation to provide medium-sized, cell-permeable ligands (28–30). Among the most common design approaches is the stabilization of α-helical peptide conformations via inter-side chain crosslinks (30–32). In particular, the design of so-called hydrocarbon-stapled peptides has seen broad application (32,33). In this setup, macrocyclization is conducted between a pair of α-methylated, non-natural amino acids featuring terminal alkene side chains. Through ring-closing metathesis, these alkenes are connected to form an intramolecular crosslink (staple) which supports the helical fold (34,35). So far, hydrocarbon-stapled peptides have not been used to target RNA and it is unclear if such structures are suitable for RNA-targeting. Also, questions remain over the general applicability of peptidomimetics as RNA-binding ligands.

Herein, we describe the design of hydrocarbon-stapled peptides derived from the *tomato aspermy virus* 2b (TAV2b) protein, a viral suppressor of RNA silencing (36). Using a reported crystal structure of TAV2b bound to ds siRNA (37), we identified a mainly α-helical 33-mer peptide that binds ds RNA with moderate affinity. To constrain the α-helical conformation, we developed a series of mono- and double-stapled peptides among which we identified a protease-stable and cell-permeable double-stapled analog with high affinity for a palindromic siRNA. Notably, we also observe binding of this stapled peptide to the oncogenic microRNA-21 duplex (miR-21) and its precursor, pre-miR-21, which both share structural features with siRNA. In biochemical assays, we observe that pre-miR-21 binding inhibits Dicer-mediated maturation.

## MATERIALS AND METHODS

### Abbreviations

Fmoc: fluorenylmethyloxycarbonyl; SPPS: solid-phase peptide synthesis; EMSA: electrophoretic mobility shift assay; EDTA: ethylenediaminetetraacetic acid; PBS: phosphate buffered saline (137 mM NaCl, 2.7 mM KCl, 10 mM Na_2_HPO_4_, 2 mM KH_2_PO_4_, pH 7.4); TFA: trifluoroacetic acid; ACN: acetonitrile; DMSO: dimethyl sulfoxide; HEPES: 4-(2-hydroxyethyl)-1-piperazineethanesulfonic acid; FCS: fetal calf serum; qRT-PCR: quantitative reverse transcription polymerase chain reaction; PAGE: polyacrylamide gel electrophoresis.

### Oligonucleotides

The sequences and names of all oligonucleotides used in this study are listed in Supplementary Table S1. High-performance liquid chromatography (HPLC)-purified oligonucleotides were purchased from *Eurofins Genomics* and *NOXXON Pharma*. Transfer-RNA (t-RNA) from baker's yeast was purchased from *Hoffmann La Roche*. For quantification, the ultraviolet (UV) absorbance of the oligonucleotides was measured in the buffer of the corresponding experiment using a V-550 ultraviolet/visible (UV/Vis) spectrophotometer (*Jasco*). Respective concentrations were calculated with a molar extinction coefficient at *λ* = 260 nm, determined according to the nearest-neighbor model using published parameters for oligonucleotides (38–42). RNA duplexes were heated to 95°C for 10 min and slowly cooled to room temperature (RT) for 1 h prior to experiments.

### Solid-phase peptide synthesis

Peptides were synthesized according to standard Fmoc-chemistry for SPPS (43) using PyBOP (benzotriazol-1-yl-oxy-tris-pyrrolidino-phosphonium hexafluorophosphate), HCTU (O-(6-chlorobenzotriazol-1-yl)-N,N,N′,N′-tetramethyluronium hexafluorophosphate), Oxyma (ethyl cyano(hydroxyimino)acetate) and COMU ((1-cyano-2-ethoxy-2-oxoethylidenaminooxy)dimethylamino-morpholino-carbenium hexafluorophosphate). For more detailed information about peptide synthesis and characterization see Supplementary Methods, Supplementary Table S2 and Figures S25–S30.

### Electrophoretic mobility shift assay

Electrophoretic mobility shift assays (EMSAs) were performed using a Bio-Rad Mini-Protean gel system paired with a direct current (DC) power source (PowerPac™ HC, Bio-Rad). Typically, 6 μl solutions containing ds RNA (*c* = 3 μM) and fluorescein-labeled peptide (*c* = 6 μM) were incubated in binding buffer (10 mM Tris (pH 8.0), 0.5 mM EDTA, 12.5% glycerol and 150 mM KCl) at 4°C for 1 h. After incubation, bound RNA was resolved from peptide-free RNA using 10% non-denaturing polyacrylamide gels at 120 V in running buffer (25 mM Tris (pH 8.0), 200 mM glycine, 20 mM NaCl) at 4°C for 1 h. Visualization of fluorescent gel-bound species was conducted using a Typhoon Trio + fluorescence scanner (GE Healthcare Life Sciences). For nucleic acid visualization, gels were stained using 2 μl of SYBR™ gold nucleic acid gel dye (Molecular Probes) in 20 ml of TAE buffer (40 mM Tris, 1 mM EDTA, 20 mM acetic acid) for 45 min at RT before being visualized using a FluorChem™ gel documentation system (Alpha Innotech). To assess **B3** binding to different ds RNA sequences (Supplementary Figure S20), 6 μl solutions containing hairpin RNA (*c* = 1 μM) and fluorescein-labeled **B3** (*c* = 2 μM) were incubated in binding buffer (TAE and 10% glycerol) at RT for 1 h, and then resolved using 15% non-denaturing polyacrylamide gels (acrylamide/bis-acrylamide (19:1) in TAE) at 150 V in running buffer (TAE) at 4°C for 1.5 h. Visualization of fluorescent gel-bound species was conducted using a Bio-Rad ChemiDoc (Bio-Rad). Subsequently, gels were stained with SYBR™ gold nucleic acid gel dye (Invitrogen) as described above and visualized using the same imaging system. To assess **B3** binding to pre–miR-21 (Supplementary Figure S24), the RNA was prepared at a fixed concentration (*c* = 1 μM) and incubated with varying concentrations of fluorescein-labeled **B3** (*c* = 0–5 μM) in binding buffer (TAE and 10% glycerol) at RT for 1 h. After incubation, bound nucleic acid complexes were resolved using 15% non-denaturing polyacrylamide gels (acrylamide/bis-acrylamide (19:1) in TAE) at 150 V in running buffer (TAE) at 4°C for 1.5 h, followed by visualization and staining with SYBR™ gold nucleic acid gel dye (Invitrogen).

### Isothermal titration calorimetry

Isothermal titration calorimetry (ITC) was conducted using a MicroCal VP-ITC (Malvern Panalytical). Before measurement, oligonucleotide and peptide samples were dissolved in PBS and degassed using a ThermoVac (Malvern Panalytical) and heated to 30°C. *N*-terminally acetylated peptides (*c* = 3–12 μM) were then transferred into the sample cell and oligonucleotides (*c* = 12–72 μM) were transferred into the syringe. 35 Injections per measurement were performed at 30°C (8 μl injection volume, 2 s injection time, 180 s spacing, high feedback mode) with an initial delay of 60 s and a stirring speed of 307 rpm. Measurements were performed in triplicate. Using the MicroCal LLC ITC software (Origin; OriginLab Corporation), the heat associated with each injection was calculated by integrating the area under the curve in microcalories per second [μca/s] versus time [min] for each injection and then normalized to concentration. A ‘single set of identical binding sites’ model was used to fit the binding curves from which thermodynamic binding parameters (Δ*G*, Δ*H*, Δ*S*, *N* and *K*_d_) were obtained.

### Circular dichroism spectroscopy and *T*_m_ determination

Circular dichroism (CD) spectra of *N*-terminally acetylated **wt33** and ds RNA were recorded with a Jasco J-1500 spectropolarimeter (*Jasco*) equipped with a programmable Peltier thermostat in a stoppered quartz cuvette (10 mm; Hellma). Samples of pal-RNA (*c* = 2 μM) and **wt33** (*c* = 4 μM) were prepared in buffer (10 mM sodium phosphate, pH 7.4, 100 mM NaCl). For each sample, 10 CD spectra were measured between *λ* = 200 and 350 nm with continuous scan mode (1 mdeg sensitivity, 1.0 nm resolution, 1.0 nm bandwidth, 2 s integration time, 100 nm·min^−1^ scan rate). Obtained spectra were averaged and then subtracted from a reference buffer spectrum. CD data were normalized to oligonucleotide strand concentration using Formula (1):

(1) $$\begin{equation*}\varepsilon l - \varepsilon r = {\rm{\Delta }}\varepsilon = \frac{\theta }{{32980 \cdot c \cdot l}}\end{equation*}$$

where *θ* = observed ellipticity (mdeg), *c* = DNA strand concentration (mol·l^−1^) and *l* = path length (cm).

Melting temperature (*T*_m_) determination was conducted using the same instrumentation and sample preparation, where ellipticity (*θ* = 267 nm) was measured by ramping the temperature from 15–90°C (4°C·min^−1^ ramp, ±0.05°C equilibration tolerance, 6 s delay after equilibration). Data points were recorded every 0.5°C. Raw data was normalized as described above and *T*_m_-values were determined using the CDpal program (44) before being plotted in Prism 5.0 (GraphPad).

Spectra of fluorescein-labeled peptides were recorded with a J-715 circular dichroism spectrometer (Jasco) using a stoppered quartz cuvette (1 mm; *Hellma*). Peptide samples (*c* = 75 μM) were prepared in buffer: 10 mM disodium hydrogen phosphate, pH 7.4. For each sample, 10 CD spectra were measured between *λ* = 190 and 260 nm with continuous scan mode (10 mdeg sensitivity, 1.0 nm resolution, 1.0 nm bandwidth, 2 s integration time, 50 nm/min scan rate). Obtained spectra were averaged, then subtracted from a reference buffer spectrum and smoothed using a fast Fourier transform filter (FFT filter). The mean residue ellipticity (*Θ*) (deg·cm^2^·dmol^−1^·10^4^) was calculated using Formula (2) and plotted against wavelength (*λ*) (nm). The ratio of secondary structure elements was calculated with CDNN using the PEPFIT set of reference spectra for secondary structure determination (45,46):

(2) $$\begin{equation*}{\left[ \Theta \right]_{\rm m}} = \frac{{\rm{\Theta }}}{{c \cdot d \cdot 10\,000}}\end{equation*}$$

$$\begin{equation*}{\rm{Given\;that}}:\;c = \frac{{{c_{\rm g}}}}{{{\rm MRW}}}\;,\;{\rm{with\;}}{\rm MRW} = {\rm{}}\frac{M}{N}\end{equation*}$$

where [*Θ*]_m_ = mean molar ellipticity per peptide bond (degree·cm^2^·dmol^−1^·10^4^), *Θ* = measured ellipticity (degree), *d* = optical path length (cm), *c*_g_ = mass concentration (g·l^−1^), MRW = mean residue weight, *M* = molar mass of the peptide (g·mol^−1^) and *N* = number of peptide bonds.

### Protease stability assay

Protease stability assays were performed in analogy to a previously published protocol (47). Stocks of fluorescein-labeled peptides were prepared to a concentration of 55.5 μM in assay buffer (10 mM Tris, pH 8.0, 150 mM NaCl) and separately stocks of proteinase K also in assay buffer (*c* = 100 μg·ml^−1^). To begin each experiment, peptide and proteinase K stocks were mixed (final concentrations: *c* = 10 μg·ml^−1^ proteinase K, *c* = 50 μM fluorescein-labeled peptide; final volume = 500 μl) and incubated at room temperature. At each time point, 50 μl of the reaction mixture was quenched by adding 100 μl of TFA/ACN/H_2_O (v/v/v, 1:49:49) and subsequently incubated for 2 min on ice. Following centrifugation (15 min, 13200 rpm, 4°C), samples (45 μl) were monitored by analytical HPLC using a linear gradient of 10–60% HPLC solvent B over 20 min. The amount of remaining peptide was quantified via absorbance at *λ* = 210 nm (intact peptide (%)) and plotted against time (*t*) (h). Half-life (*t*_1/2_) was calculated by fitting the data to an exponential decay using the software Prism 5.0 (GraphPad).

### Peptide uptake

2 × 10^6^ K562 cells were plated per well in 48-well plates in presence of fluorescein-labeled peptides (*c* = 1 μM, 0.5% DMSO) in a total volume of 300 μl. After 90 min, cells were treated with proteinase K (2 μg·ml^−1^) for 10 min to remove extracellular peptides. Cells were extensively washed and total uptake of labeled peptide was measured by flow cytometry (Cytoflex, Beckman Coulter with the CytExpert software version 2.3). For each peptide, 20 000 events per sample were collected with >85% viable cells (forward/side scatter) that were considered for analysis using a 525/40 nm filter. DMSO was used as a control to distinguish general DMSO-related and peptide-specific effects. Each experiment was performed in triplicate.

### Modeling of wt33 peptide and RNAs

Structural models of pre-miR-21 RNA were generated by a stepwise modeling approach (48). Briefly, the initial model was generated with the ModeRNA server (49) using the loop region from the nuclear magnetic resonance (NMR) structure (PDB ID: 5UZT) (50) as the template. It was then refolded with SimRNA (51), a method for simulations of RNA folding which uses a coarse-grained representation and relies on the Monte Carlo method for sampling the conformational space. SimRNA employs a statistical potential to approximate the energy using additional restraints on the secondary structure taken from miRBase database (52). Finally, the best-scoring model from SimRNA was optimized in the full-atom representation with QRNAS (53). The starting model of the **wt33** peptide was generated based on the crystal structure of the TAV2b/siRNA complex (PDB ID: 2ZI0) (37). Models of the peptide/RNA complexes were generated using SimRNP, an extension of SimRNA (51), which uses a representation of protein structures similar to that in REFINER (54) and an energy function derived from experimentally solved RNA and RNP structures. pre-miR-21 was simulated in the presence of triple copies of the **wt33** peptide.

For best-scored complexes generated from SimRNP simulations, all-atom molecular dynamics (MD) simulations were carried out for pre-miR-21 with complexes of three copies of **wt33** peptide using the Amber 18 program suite (55). The starting structures for the simulation were prepared using tleap in a truncated octahedral box of 10 Å allowance along with TIP3P water model (56). A combination of two force-fields, Amber ff14sb force-field for proteins (57) and the χOL3 force-field for RNA (58,59), was used to describe the biomolecules during the simulations. A two steps energy-minimization (each with 10 000 cycles) was performed with and without restraints, respectively, followed by heating, density equilibration, and short runs of equilibration. The heating was done from 100 to 300 K for 500 ps with restraints on the entire structure and the density equilibration was performed for 500 ps, also with restraints on the entire structure. The equilibration of the structures was run for four short rounds. The first three rounds of equilibration were run for 200 ps each with the main chain atoms constrained. The final round of equilibration was performed for 2 ns, followed by the production run for 100 ns. Sander was used for performing the minimization steps and the following steps were performed using the CUDA version of PMEMD from the Amber program suite (60,61).

### Pull-down

2 × 10^6^ K562 cells were incubated with *N*-terminally biotinylated peptides (*c*(peptide) = 10 μM, *t* = 2.5 h) for uptake on a rotating wheel at 37°C. The cells were extensively washed in PBS and lysed in lysis buffer (25 mM HEPES, pH 7.5, 150 mM NaCl, 0.05% IGEPAL CA-630, 1 mM EDTA) supplemented with 0.5 U/μl RNAse inhibitor (Superase-in, ThermoFisher). The lysate was cleared by centrifugation at 21 000 g at 4°C, an aliquot of the supernatant was removed and re-suspended in Trizol, while the remaining was incubated with streptavidin-coupled magnetic beads (Dynabeads M280, ThermoFisher) on a rotating wheel (2 h, 4°C). After magnetic separation and washing (3× in lysis buffer), the beads were re-suspended in 20 μl lysis buffer, re-suspended in Trizol (ThermoFisher), and 400 ng t-RNA was added per sample as a carrier. RNA was extracted from Trizol using the standard protocol and the qRT-PCR was performed according to a previously published protocol (62) using the primers h-miR21-3p-FW (CAGCAACACCAGTCGATG), h-miR21-3p-RV (GGTCCAGTTTTTTTTTTTTTTTACAG), h-miR21-5p-FW (GCAGTAGCTTATCAGACTGATG), h-miR21-5p-RV (GGTCCAGTTTTTTTTTTTTTTTCAAC) in a CFX96 real-time machine (Bio-Rad). The relative enrichment was calculated as a ratio of copies in the immunoprecipitate over input normalized to the enrichment of the control peptide **D1**.

### Confocal microscopy

Non-adherent K562 cells were counted and collected in 1.5 ml Eppendorf tubes at 30 000 cells per tube. After a wash, cells were re-suspended in 200 μl of phenol-red free media containing 5% FCS and fluorescein-labeled peptides (*c*(peptide) = 1 μM, *t* = 30 min) in the Eppendorf tubes at 37°C with vertical rotation to avoid cell pelleting. After incubation, cells were washed once with phenol-red free media, re-suspended again in the same media, and transferred into the wells of an 8-chamber μ-slide (Ibidi). Cells were immediately imaged at 37°C with an SP5 Laser Scanning Confocal Microscope (Leica). Fluorescein was excited with an argon ion laser at wavelength of 488 nm and fluorescence was detected in the range 505–530 nm.

### Dicer cleavage assay

To assess the inhibitory ability of **B3** and **D1** for pre–miR–21 Dicer cleavage, 10 μl solutions of each *N*–terminally acetylated peptide at varying concentrations (*c* = 10, 50, 150, 300 or 600 nM) were prepared with ds pre–miR–21 (*c* = 40 nM) in buffer (20 mM Tris hydrochloride (Tris·HCl), pH 6.8, 12.5 mM NaCl, 2.5 mM MgCl_2_, 1 mM dithiothreitol, 2% DMSO, and 0.02% bovine serum albumin (BSA)). Next, RhDicer (0.5 U·μl^−1^ solution; *Genlantis*) was diluted in buffer (20 mM Tris·HCl, pH 6.8, 100 mM NaCl, 1 mM MgCl_2_, 1 mM DTT, 5 mM β-mercaptoethanol, 10% glycerol, 0.1% Triton-X 100) to a concentration of 40 U·ml^−1^ and then added to the peptide/RNA solutions (final RhDicer concentration: 4 U·ml^−1^). The reaction solution was incubated for 150 min at 37°C, then denatured for 5 min at 70°C, and stored at 4°C. Subsequently, a 1:10 dilution in H_2_O of the reaction solution was performed and added to a Poly(A) Tailing and reverse transcription solution as previously reported (62). The samples were incubated at 42°C for 1 h before subsequent inactivation for 5 min at 95°C and cooling to 4°C. Next, qRT-PCR of each sample was performed using the primers miR21–5p–RV1 (GGTCCAGTTTTTTTTTTTTTTTCAAC), miR21-5p-FW1 (GCAGTAGCTTATCAGACTGATG), and miRNA-RT1 (CAGGTCCAGTTTTTTTTTTTTTTTVN) in a StepOnePlus™ real-time PCR system (Thermo Fisher Scientific). Briefly, 1 μl of template DNA solution was added to a solution of Luna^®^ Universal Probe qPCR Master Mix (New England Biolabs) with a final primer concentration of 125 nM in 20 μl per sample. Samples were initially denatured for 5 min at 95°C followed by 45 temperature cycles of 15 s at 95°C and 30 s at 66°C. The quantification was done using miR-21-5p standard curves. To visualize the inhibitory potential of each peptide the normalized abundance of mature miR-21 (%) is plotted against the peptide concentration (nM).

## RESULTS

### Design of TAV2b-derived stapled peptides

**Figure 1.**
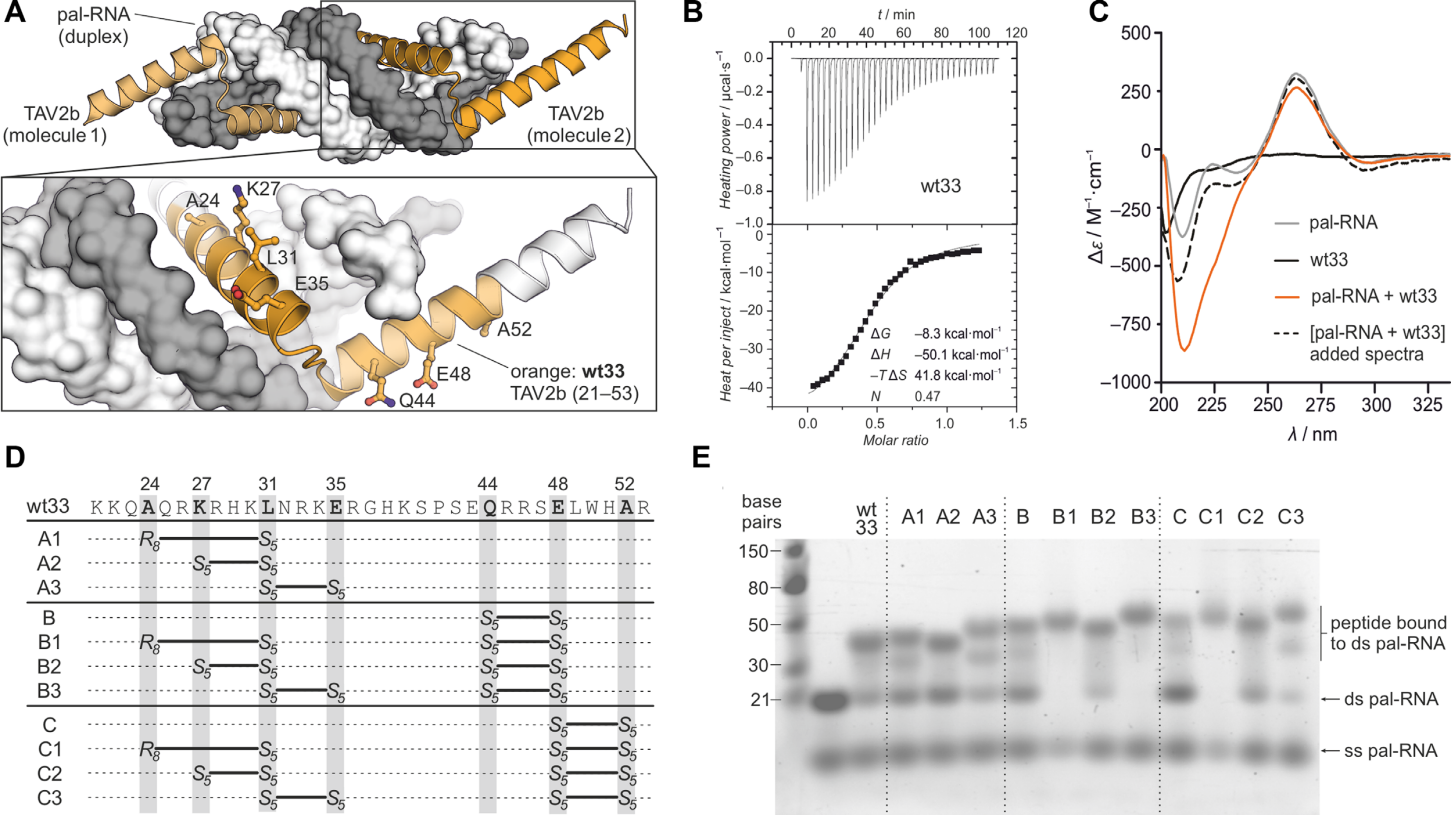
(**A**) Crystal structure (PDB ID: 2ZI0) showing two TAV2b peptides (orange) bound to pal–RNA (gray). Close-up highlights the **wt33** sequence (K21–R53, orange, cartoon representation) in complex with ds pal-RNA (gray and white, surface representation). Selected **wt33** side chains are shown in ball-and-stick representation. (**B**) Representative ITC of unlabeled **wt33** with the pal–RNA duplex (for full data see Supplementary Table S3 and Figure S5). (**C**) CD spectra of **wt33** (*c* = 4 μM), pal-RNA (*c*(duplex) = 2 μM), spectra of pal-RNA (*c*(duplex) = 2 μM) with **wt33** (*c* = 4 μM) in solution (pal-RNA + **wt33**), and the sum of the two individual spectra ([pal-RNA +**wt33**]). Buffer: 10 mM sodium phosphate, pH 7.4, 100 mM NaCl. (**D**) Sequences of TAV2b-derived peptides highlighting macrocyclization points. (**E**) EMSA of ds pal-RNA incubated with fluorescein-labeled TAV2b-derived peptides (**A1**–**A3**, **B**–**B3**, **C**–**C3**). Experiments employed 10% native PAGE (*c*(RNA) = 3 μM, *c*(peptide) = 6 μM). Running buffer: 25 mM Tris, pH 8.0, 200 mM glycine, 20 mM NaCl. For full gels including fluorescein readout, see Supplementary Figure S6 (ds: double-stranded, ss: single-stranded).

Belonging to the *Bromoviridae* family of plant viruses, the *Tomato aspermy virus* (TAV) is a common infectious agent known to cause mosaic symptoms, necrosis and deformation in tomato and chrysanthemum plants. As a positive-sense, single stranded RNA virus, the TAV genome is tripartite, where RNA 1 encodes for proteins involved in replication, RNA 3 encodes for capsid proteins, and RNA 2 encodes for the protein TAV2b, a viral suppressor of RNA silencing (63). TAV2b consists primarily of a helix-loop-helix motif which binds double-stranded (ds) A–form RNA in a hook-like manner (Figure 1A and Supplementary Figure S1) (37). Both helices sit within the RNA major groove with the majority of contacts formed with the RNA backbone resulting in the sequence-independent recognition of ds RNA structures (37). In the crystal structure, two TAV2b molecules are bound to a palindromic RNA duplex (pal-RNA; single strand sequence: AGACAGCAUUAUGCUGUCUUU). We aimed to exploit the unique structural properties of TAV2b to design RNA-targeting peptides. To identify the minimal peptide sequence of TAV2b, we synthesized a series of fragments containing both helices but with different lengths (Supplementary Figure S2). These truncated peptides were designed excluding the coiled-coil region at the N-terminus of TAV2b (Supplementary Figure S1) to avoid undesired peptide self-assembly and aggregation.

The binding of each fluorescein-labeled peptide to the pal–RNA duplex was then assessed using an electrophoretic mobility shift assay (EMSA). From the truncation series, a 33-mer peptide (**wt33**, orange, Figure 1A) ranging from K21 to R53 was the shortest analog to retain RNA-binding (Supplementary Figure S2). Isothermal titration calorimetry (ITC) was next used to study the interaction of an unlabeled version of **wt33** with ds pal–RNA, revealing a 2:1 binding stoichiometry i.e. two peptides recognizing one pal-RNA duplex and moderate binding affinity (*K*_d_ = 1.19 μM; Figure 1B), which is about 16-fold lower than the one of the entire RNA-binding domain of TAV2b (amino acids 1–69, *K*_d_ = 75 nM) (37). The reported enthalpy-driven binding behavior of TAV2b was also observed for **wt33** (Δ*H* = −50 kcal·mol^−1^). Subsequently, circular dichroism (CD) spectroscopy was used to characterize unbound peptide **wt33** and pal-RNA. The spectra indicate the expected double-helical structure for pal-RNA (gray line, Figure 1C) but a mainly unstructured character for unbound **wt33** (black line). Co-incubation of pal-RNA and **wt33** (orange line, pal-RNA + **wt33**) resulted in a pronounced negative ellipticity between *λ* = 208 and 222 nm. Notably, this is not the case when the individual spectra of **wt33** and pal-RNA are simply added (black dashed line, [pal-RNA + **wt33**], Figure 1C). The spectral differences between the unbound and bound **wt33** suggest that the peptide experiences an increase in α-helicity upon RNA-binding.

To stabilize the helical conformation of the unbound peptide and thereby increase its binding affinity, a series of **wt33**-derived hydrocarbon-stapled peptides was designed. Hydrocarbon-stapling involves the introduction of two olefin-bearing, non-natural α-methylated amino acids (*S*_5_ or *R*_8_, Supplementary Figure S3) during solid-phase synthesis (43). The preferred spacing is *i*, *i* + 4 and *i*, *i* + 7 aiming at amino acid positions that are not directly involved in target binding (35). The olefin side chains are crosslinked via ring-closing metathesis providing macrocyclic peptides (Supplementary Figure S4). Based on the crystal structure of TAV2b in complex with the pal-RNA duplex, suitable amino acid positions within the **wt33** sequence (orange, Figure 1A) were identified (A24, K27, L31, E35, Q44, E48 and A52). These positions facilitate the introduction of three different crosslinks in the *N*-terminal helix (**A1**–**A3**) and two in the *C*-terminal helix (**B** and **C**). In addition to single-stapled peptides, crosslinks were combined resulting in six double-stapled peptides (**B1**–**B3** and **C1**–**C3**; Figure 1D).

Eleven stapled peptides were synthesized and their binding to ds pal-RNA assessed via EMSA (Figure 1E and Supplementary Figure S6). Peptides were labeled with fluorescein as a means to verify the formation of such peptide/RNA complexes using fluorescence visualization (Supplementary Figure S6). Due to their positive charge unbound peptides did not migrate into the gel. RNA alone appears as two bands with the major one corresponding to the pal–RNA duplex, and the weaker to ss pal–RNA. For all peptides, the appearance of one or two elevated bands was observed indicating the formation of RNA/peptide complexes which was also confirmed by a corresponding signal in the fluorescence visualization (Supplementary Figure S6). These two new bands can be associated with one and two bound peptides, respectively. Formation of these bands results in a reduction of the pal-RNA duplex band. Interestingly, ss pal-RNA shows only a low response to peptide treatment. A full disappearance of the ds pal-RNA band was only observed for peptides **B1**, **B3** and **C1** hinting at particularly potent RNA-binding (Figure 1E).

### Double-stapled peptides show enhanced helicity and affinity for ds pal-RNA

**Figure 2.**
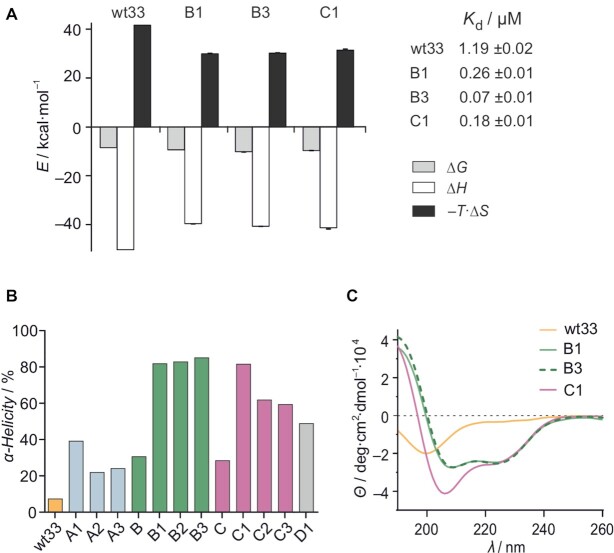
(**A**) ITC-derived thermodynamic properties of pal-RNA binding to unlabeled **wt33**, **B1**, **B3** and **C1**, including *K*_d_-values (measurements were performed in triplicate, errors account for 1σ; for full data see Supplementary Table S3 and Figure S11–S13). (**B**) Percentage of α-helical secondary structure content for each fluorescein-labeled peptide. Buffer: 10 mM sodium phosphate, pH 7.4 (for complete secondary structure distribution, see Supplementary Table S4). (**C**) Overlaid CD spectra of fluorescein-labeled **wt33**, **B1**, **B3** and **C1** (*c*(peptide) = 75 μM). Buffer: 10 mM sodium phosphate, pH 7.4.

To quantify RNA-binding of these peptides, ITC binding experiments with unlabeled analogs of **B1**, **B3** and **C1** were performed, revealing 5-, 17- and 7-fold increased affinity, when compared to linear peptide **wt33** (Figure 2A). In analogy to **wt33**, all three stapled peptides exhibit enthalpy-driven binding, though to a reduced extent (Δ*H*(**wt33**) = 5.0 versus Δ*H*(stapled peptides) = 3.9−4.1 kcal·mol^−1^). This decrease in binding enthalpy is compensated by a reduction in the entropic penalty upon binding (−*T·*Δ*S*(**wt33**) = 42 versus −*T·*Δ*S*(stapled peptides) = 30–32 kcal·mol^–1^) which likely stems from the helical pre-organization induced by stapling. Evidence of this structural pre-organization was also observed with CD spectroscopy. In solution, peptides **B1**, **B3** and **C1** display characteristic α-helical spectra. Unlike **wt33**, an enhancement in ellipticity between *λ* = 208 and 222 nm was not observed when these peptides were incubated with ds pal–RNA (Supplementary Figures S7–S9). These data indicate that stapling indeed enforced the α-helical conformation of peptides **B1**, **B3** and **C1**, thereby promoting pal-RNA duplex binding. For subsequent experiments, control peptide **D1** was included which features two hydrocarbon crosslinks within the RNA-interface and does not bind to ds pal-RNA in EMSAs (Supplementary Table S2 and Figure S10). Initially, the entire panel of fluorescein-labeled peptides including **D1** was characterized via CD spectroscopy to determine α-helicity in the unbound form (Figure 2B). Notably, all stapled peptides showed higher α-helicity than **wt33** (α-helicity = 7.3%) with the double-stapled derivatives exhibiting a stronger helical character than the mono-stapled analogs (Figure 2B). High-affinity peptides **B1**, **B3** and **C1** are among the most helical sequences (α-helicity = 82–85%) with their CD spectra showing minima at *λ* = 208 and 222 nm characteristic for α-helices (Figure 2C).

### Peptide stapling increases protease stability and cell permeability

**Figure 3.**
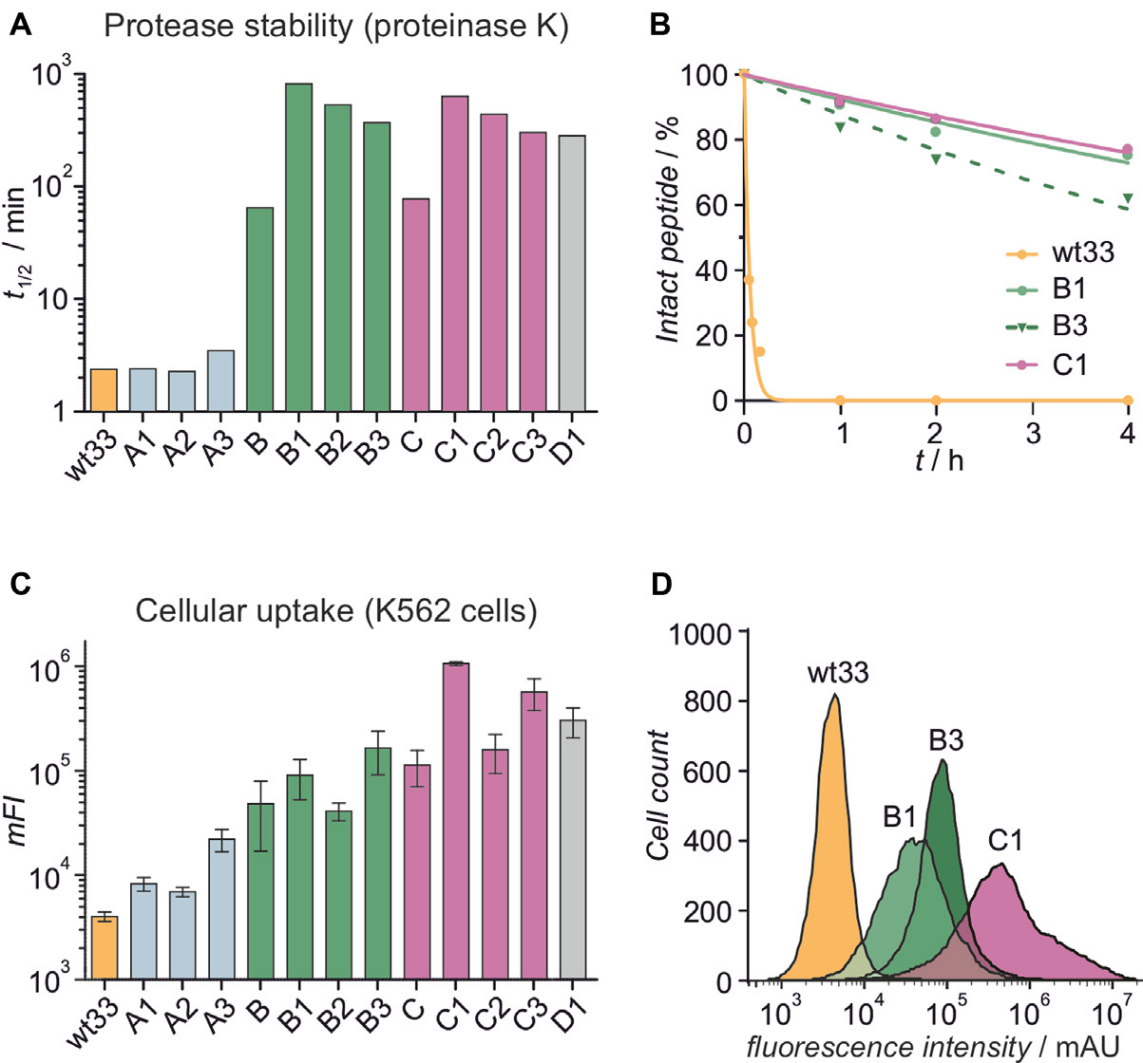
(**A**) Half-life (*t*_1/2_) values for fluorescein-labeled peptides (*c* = 50 μM) in the presence of proteinase K (*c* = 10 μg·ml^−1^). Buffer: 10 mM Tris, pH 8.0, 150 mM NaCl. (**B**) Percentage of intact fluorescein-labeled peptide remaining over time in the presence of proteinase K. (**C**) *mFI*-Values of K562 human leukemia cells treated with fluorescein-labeled peptides (*c* = 1 μM, *t* = 90 min, triplicate of measurements, errors account for 1σ). (**D**) Fluorescence intensity distribution of K562 cells exposed to fluorescein-labeled **wt33**, **B1**, **B3** and **C1**.

A protease stability assay was conducted by incubating the fluorescein-labeled peptides with proteinase K and monitoring peptide integrity by HPLC-MS to determine peptide half-life (*t*_1/2_, Figure 3A) (47). Linear peptide **wt33** experienced rapid cleavage (*t*_1/2_ = 2.4 min, Supplementary Table S5), whereas the introduction of crosslinks in the *C-*terminal helix resulted in increased stability (**B** and **C**, *t*_1/2_ = 65 and 78 min, respectively) while stapling of the *N-*terminal helix did not improve protease resistance (**A1**–**A3**, *t*_1/2_ ≈ 3 min). A combination of both *N-* and *C-*terminal staples yielded the most stable peptides, with double-stapled peptides **B1** and **C1** experiencing the strongest increase in tolerance towards proteinase K (**B1**: 354-fold and **C1**: 265-fold increased stability compared to **wt33**, Figure 3B).

The cellular uptake of the peptides was assessed using K562 human leukemia cells and flow cytometry as readout (64). Cells were treated with fluorescein-labeled peptides (*c* = 1 μM, *t* = 90 min) which was followed by a stringent wash protocol including tryptic digestion. Flow cytometry-based mean fluorescence intensities (*mFI*) were then determined (Figure 3C). Compared to **wt33** (*mFI* = 4031 AU) all stapled peptides caused increased fluorescence intensities indicative of increased cellular uptake. In line with trends observed for α-helicity (Figure 2B) and proteolytic stability (Figure 3A), the double-stapled peptides displayed an increased cellular uptake when compared to their mono-stapled analogs. Overall, double-stapled peptide **C1** showed the highest increase in uptake relative to **wt33** (264-fold; Figure 3D). Despite its lower affinity for pal–RNA, considerably enhanced cellular uptake was also observed for control peptide **D1**. Based on these observations and their high affinity for ds pal-RNA, we decided to use double-stapled peptides **B3**, **C1** and control **D1** for further investigations. For that reason, cellular uptake of these peptides was investigated by live cell confocal microscopy confirming their cell permeability (Supplementary Figure S14).

### Double-stapled peptides bind ds miR-21 structures

The protein TAV2b was reported to preferably bind ds RNA and we therefore assessed the binding of **B3** and **C1** to alternative nucleic acid structures using EMSA. Analogous to TAV2b, both stapled peptides did not bind single-stranded RNA (ss RNA) or the ds DNA analog of pal-RNA (Supplementary Figures S15 and S16). Notably, we also did not observe binding of **B3** and **C1** to a mixture of yeast transfer RNAs (t-RNAs) indicating that peptide binding indeed requires accessible RNA duplex structures. Although not reported for TAV2b, other viral suppressors of RNA silencing are known to bind microRNAs (miRNAs) (65). Similar to siRNAs, miRNAs are short non-coding RNAs that are incorporated into the RISC complex leading to the blockage or degradation of complement mRNA (66). miRNAs arise from long stem-loop-bearing RNAs in the nucleus (pri-miRNAs) which are processed and exported as shorter hairpin structures, so-called pre-miRNAs. Pre-miRNAs are further processed by the endoribonuclease Dicer resulting in the formation of mature ds miRNAs (67).

**Figure 4.**
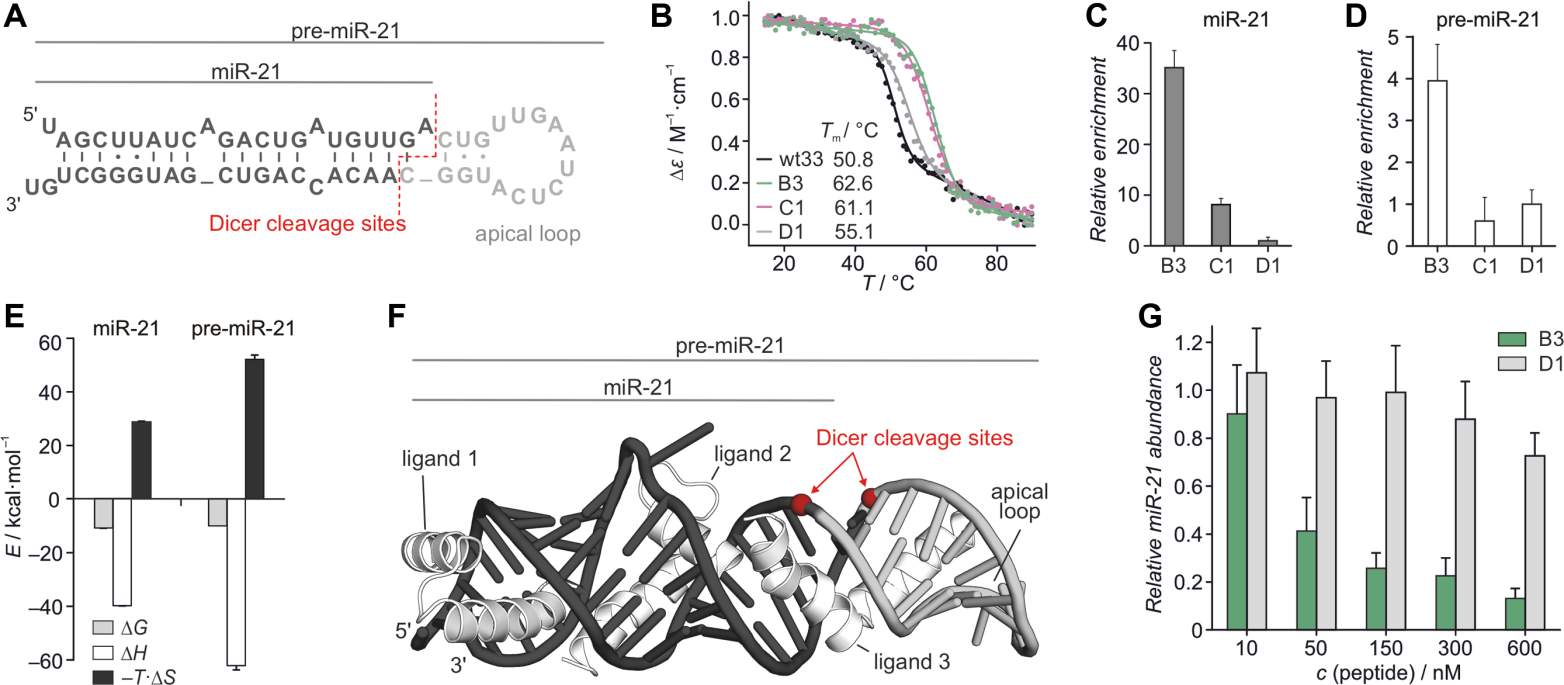
(**A**) Sequence of the pre-miR-21 hairpin (black/gray) with the miR-21 duplex shown in black. Locations of Dicer cleavage sites are indicated (red). (**B**) Melting profiles of miR-21 in the absence and presence of double-stapled peptides **B3**, **C1** and **D1** (*λ* = 267 nm, *c*(miR-21) = 2 μM, *c*(peptide) = 4 μM. Buffer: 10 mM sodium phosphate pH 7.4, 100 mM NaCl). (**C**, **D**) Enrichment of miR–21 and pre–miR–21, respectively, after treatment of K562 cells with the biotinylated peptides **B3**, **C1** and **D1** and subsequent streptavidin-mediated selection of peptide-bound RNA relative to total RNA after cell lysis (triplicate of measurements, errors account for 1σ). (**E**) ITC-derived thermodynamic properties of **B3** binding to miR–21 and pre–miR–21 (measurements were performed in triplicate; for full data see Supplementary Table S3 and Figures S22 and S23). (**F**) SimRNP model of a complex involving three **wt33** peptide ligands (white, cartoon representation) and pre-miR–21 (black/gray, cartoon representation). Dicer cleavage sites are indicated (red). Coordinates of the model can be found as supplementary data. (**G**) Relative miR-21 abundance after Dicer processing of pre-miR-21 in the presence and absence of **B3** or **D1** using qRT-PCR (relative to absence of peptide control; *c*(pre-miR-21) = 40 nM, *c(*Dicer) = 4 U·ml^−1^; triplicate of measurements, errors account for 1σ).

Given the sequence-independent nature of TAV2b-binding, we suspected double-stapled peptides **B3** and **C1** to also bind miRNA structures. To test this hypothesis, we chose ds miRNA-21 (miR-21) as an example. miR-21 represents the first miRNA species which was shown to directly influence oncogenic progression. Upregulation of miR-21 is common to most types of cancer and is implicated in the suppression of genes associated with cell growth and differentiation such as *PDC4*, *RECK, TPM1* and *PTEN* (68). Similar to pal-RNA, the miR-21 duplex is comprised of two short RNA strands but features a central one-nucleotide bulge as well as a single mismatched base pair (black, Figure 4A) (68,69). In pre-miR-21, the two RNA strands (black) are connected by a partially self-complementary sequence (gray, Figure 4A) resulting in the adoption of a hairpin-like structure (50). Initially, EMSAs were used to investigate the ability of fluorescein-labeled **B3**, **C1** and **D1** to bind ds miR–21. While we observed the formation of peptide/RNA complexes for **B3** and **C1**, control peptide **D1** did not show binding (Supplementary Figures S17–S19). It has been reported that nucleic acid duplexes can be stabilized by bound ligands (70). Therefore, thermal denaturation experiments were performed to test the effect of peptide binding on the stability of the miR-21 duplex. Indeed, the incubation of miR-21 (*c* = 2 μM) with unlabeled peptides **B3** or **C1** (*c* = 4 μM) resulted in increased melting temperatures (Δ*T*_m_ > 10°C), while **D1** only showed a mild effect on duplex stability (Figure 4B).

Having confirmed ds miR-21-binding of **B3** and **C1**, we assessed their binding to endogenous miR-21 in a cellular context. For that purpose, pull-down experiments were performed in which K562 human leukemia cells were initially incubated with biotinylated analogs of **B3**, **C1** or **D1** (*c* = 10 μM, *t* = 180 min). Subsequently, cells were rigorously washed, lysed and finally lysates were treated with streptavidin beads to capture the peptides in complex with RNA. After extraction of RNA from the beads, the amount of miR-21 was evaluated by quantitative reverse transcription polymerase chain reaction (qRT-PCR). In this experimental setup, peptide cell permeability, bio-stability and RNA-binding contribute to pull-down efficiency. For peptides **B3** and **C1**, we observed enrichment when compared to control peptide **D1** (Figure 4C), which was particularly pronounced for **B3** (35-fold). Given the structural similarities between ds miR–21 and its hairpin precursor, pre-miR–21 (Figure 4A), we also tested binding of fluorescein-labeled **B3** and **C1** to pre-miR–21 initially using EMSA. For both peptides, EMSA indicated pre–miRNA-binding while this was not the case for control peptide **D1** (Supplementary Figures S17–S19). In pull-down experiments, however, we only observed enrichment of pre-miR-21 when using biotinylated **B3** (4-fold, Figure 4D), though to a lower extent than for miR-21 (35-fold, Figure 4C). In line with its lower miR-21 pull-down efficiency, **C1** did not show detectable pre-miR-21 enrichment. Based on the good performance of **B3** in the miR-21 and pre-miR-21 pull-down experiments, this double-stapled peptide was selected for further investigations.

While **B3** appears to be specific for the binding of ds RNA (over ss RNA, ss DNA and ds DNA), the high affinity binding of pal-RNA, miR-21 and pre-miR-21 suggests sequence-independent recognition of ds RNA structures. This is in line with the binding characteristics of the parent protein TAV2b (37). To test the sequence context more broadly, we investigated the binding of **B3** to a set of five ds RNA hairpins, composed of different, complementary 19 base pair stems bridged via a fixed 6 nucleotide loop (Supplementary Figure S20). In EMSA experiments, **B3** showed binding to each hairpin, resulting in the occurrence of the expected two new bands (peptide/RNA 1:1 and 2:1, Supplementary Figure S20) indicating indeed sequence-independent binding. The parent protein, TAV2b, recognizes ds RNA through a series of hotspot arginine residues (R26, R33 and R46) on both of the two α–helices thereby recognizing the major groove of the RNA duplex (Supplementary Figure S21). To test whether **B3** uses an analogous binding mode, we synthesized a panel of **B3** variants in which these arginine residues were substituted by glutamic acid (Supplementary Figure S21). Notably, EMSA experiments confirm a severe loss in binding affinity for all variants effectively abolishing **B3** binding to ds miR-21 (Supplementary Figure S21). This suggests a binding mode for **B3** similar to TAV2b.

Next, we used ITC to quantify binding of **B3** to miR-21 (Figure 4E). These measurements revealed a high complex stability (*K*_d_ = 18 ± 2 nM) exceeding the binding affinity of **B3** for the pal-RNA duplex (*K*_d_ = 70 ± 1 nM, Supplementary Table S3). Binding was again enthalpy-driven with the expected binding stoichiometry of about 2:1 (**B3**/miR-21, *N* = 0.43). We also used ITC to investigate **B3** binding to pre-miR-21, revealing a high affinity (*K*_d_ = 75 ± 8 nM) though with a changed binding stoichiometry indicative of 3:1 or 4:1 binding (**B3**/pre-miR-21, *N* = 0.28; Supplementary Table S3). Given the elongated stem region in pre-miR-21, we suspected the binding of at least one additional **B3** molecule. To further investigate the binding stoichiometry, EMSA experiments were performed using a fixed pre-miR-21 concentration with increasing amounts of **B3** (Supplementary Figure S24). Here, we observed the concentration-dependent formation of up to three new distinct bands with saturation occurring after the formation of the third band (Supplementary Figure S24). These findings strongly suggest the formation of a 3:1 peptide/RNA complex. To test the general feasibility of such a complex, replica-exchange Monte Carlo simulations of the unmodified peptide sequence with pre-miR–21 were performed using SimRNA and SimRNP computational methods that allow for flexible modeling of protein/RNA complexes (51). According to the simulation, pre-miR–21 can indeed accommodate up to three peptide sequences (Figure 4F), two of them (ligand 1 and 2) in the area of the miR–21 duplex region (black) while the third peptide (ligand 3) mainly contacts the apical loop (gray, Figure 4F). Notably, ligand 3 is predicted to bind the region that is recognized by the endoribonuclease Dicer.

Dicer binding of pre-miR-21 eventually results in strand cleavage at two sites (A29 and C46; red, Figure 4F) yielding mature ds miR-21 (black, Figure 4F). Based on the presumably overlapping binding sites of our peptides and Dicer, we were interested to test how high-affinity double-stapled peptide **B3** affects pre-miR–21 maturation. For this purpose, a biochemical maturation assay was established where recombinant Dicer (*c* = 4 U·ml^−1^) was used to process pre-miR–21 (*c* = 40 nM) into mature miR-21. A qRT-PCR detection protocol (62) was adapted to monitor miR–21 formation. Notably, pre-miR-21 maturation by Dicer was inhibited in a concentration-dependent manner by double-stapled peptide **B3** (green, Figure 4G) while control peptide **D1** (gray, Figure 4G) showed only small effects. Half-maximal Dicer inhibition by **B3** is about 50 nM which is in the concentration range of pre-miR–21 (*c* = 40 nM) in this assay. This indicates efficient competition with Dicer under these conditions.

## DISCUSSION

The design of RNA-targeting ligands remains an outstanding challenge. Antisense oligonucleotides represent the most established entities and while possessing high affinity and selectivity they tend to suffer from delivery issues (71). Alternative approaches to target RNA make use of extended or multivalent ligands derived from large-scale screening approaches or from natural products such as aminoglycosides (72–74). However, these approaches involve complicated development processes since only limited structural information is available. With the growing number of strategies to derive protein mimetics (75), new avenues towards the design of potent nucleic acid binders are being explored (76–79). Herein, we applied a structure-based design approach to generate constrained peptide mimetics of the RNA-binding protein TAV2b. As an initial starting point, we identified a 33-mer TAV2b-derived peptide (**wt33**) as the minimal sequence for ds RNA binding. Based on this peptide, a panel of hydrocarbon-stapled peptides was designed aiming at the stabilization of the RNA-bound α–helical conformation of TAV2b. The obtained stapled peptides show enhanced α-helicity, protease stability, and cell permeability, particularly pronounced for double-stapled analogs. From that panel, double-stapled peptide **B3** was investigated in most detail. It binds palindromic siRNA with high affinity but not single-stranded and transfer RNA or ds DNA. Notably, **B3** also shows high affinity for additional double-stranded RNA structures including miR-21 and its precursor pre-miR-21. Binding of **B3** to pre-miR-21 was also shown to inhibit RNA maturation by the endoribonuclease Dicer in a biochemical assay.

Double-stapled peptide **B3** represents a structural mimetic of the RNA-binding TAV2b protein. Recapitulating central features of TAV2b binding, **B3** engages ds RNA in a sequence-independent and enthalpy-driven manner presumably featuring an analogous interaction pattern. In analogy to TAV2b, complex formation with ds RNA is strongly preferred over ss RNA, likely due to the absence of a defined binding groove in the unpaired and highly flexible ss RNA chain. **B3** also exhibits a strong preference for ds RNA over ds DNA. This can be explained as ds DNA tends to exist in the B-form possessing wider and shallower major grooves than A-form RNA thereby preventing analogous interactions (80). The α-helical conformation of TAV2b is central to its high affinity for ds RNA. Our studies indicate that a truncated version of TAV2b (**wt33**) is unstructured in its free form but shows high α-helicity upon binding. This can be expected to result in a considerable entropic penalty and likely contributes to **wt33**’s relatively low affinity. Introduction of chemical constraints to stabilize the α-helical conformation, as seen for **B3**, consequently increases binding affinity for ds RNA. Highest binding affinities were obtained when both α-helices had been stabilized. However, despite the judicious placement of hydrocarbon staples opposite to the RNA-binding interface, differences in binding affinities and therefore presumably in the structural pre-organization have been observed. These differences could arise from ‘over-constraint’, where the peptide is held in a helical or intermediary conformation which is restrictive to binding the dynamic RNA interface. We have previously observed such an effect on constrained peptides targeting a transcription factor interface, where the introduction of more flexible cross-links was required to fine-tune target affinity (81).

Overall, this study highlights how RNA-binding proteins can serve as valuable starting points for the structure-based design of cell-permeable, high affinity RNA ligands, in particular when considering the growing number of available protein/RNA complex structures (8). Such design strategies can complement established protein- and nucleic acid-based targeting approaches as well as combinatorial screening efforts (18,71,82,83). Additionally, peptide-based ligands can offer new opportunities to develop probes with fine-tuned or switchable RNA-binding characteristics. Double-stapled peptide **B3** is a prime example of a modular scaffold combining bioavailability and -stability with high affinity for ds RNA major grooves. A sequence-specific recognition of ds RNA would increase the applicability of such peptides and could be achieved, e.g., via the incorporation of base-recognizing residues or the addition of modalities that feature such properties. This may involve the conjugation of antisense nucleic acids or the introduction of minor groove-targeting motifs as well as the development of RNA degrader molecules (84). Overall, **B3** represents an attractive starting point for the future design of ligands with altered RNA-recognition properties.

## Supplementary Material

gkab1149_Supplemental_Files

## Notes

Present address: Chandran Nithin, Laboratory of Computational Biology, Faculty of Chemistry, Biological and Chemical Research Centre, University of Warsaw, Warsaw 02-089, Poland.
