# Supplementary material for: Constrained peptides mimic a viral suppressor of RNA silencing
Source: Nucleic Acids Res. 2021 Dec 6;49(22):12622–33. doi: 10.1093/nar/gkab1149 (PMC8682738; doi:10.1093/nar/gkab1149)
Supplement: gkab1149_Supplemental_Files [file gkab1149_supplemental_files.zip › TAV2b_mimetic_SI_production.pdf]

Supplementary Information

Constrained peptides mimic a viral suppressor of RNA silencing

Arne Kuepper et al.

Table of Contents

1. Supplementary Methods **2**

1.1 Abbreviations 2

1.2 Solid-phase peptide synthesis 2

2. Supplementary Tables **5**

Table S1: 5

Table S2: 6

Table S3: 7

Table S4: 8

Table S5: 9

3. Supplementary Figures **10**

4. Supplementary References **35**

1. Supplementary Methods

1.1 Abbreviations

Fmoc: fluorenylmethoxycarbonyl; NMP: *N*-methyl-2-pyrrolidone; DCM: dichloromethane; COMU: (1-cyano-2-ethoxy-2-oxoethylidenaminooxy)dimethylamino-morpholino-carbenium hexafluorophosphate; OXYMA: ethyl cyano(hydroxyimino)acetate; DIPEA: *N*,*N*-diisopropylethylamine; PyBOP: benzotriazol-1-yl-oxy-tris-pyrrolidino-phosphonium hexafluorophosphate; NMM: *N*-methyl morpholine; Ac_2O: acetic anhydride; DMF: *N*,*N*-dimethylformamide; HCTU: 2-(6-chloro-1-H-benzotriazol-1-yl)-1,1,3,3-tetramethylaminium hexa-fluorophosphate; DCE: 1,2-dichloroethane; DMSO: dimethyl sulfoxide; FITC: fluorescein-5-isothiocyanate (isomer I); TIPS: triisopropylsilane; EDT: 1,2-ethanedithiol; TFA: trifluoroacetic acid; ACN: acetonitrile; FA: formic acid.

1.2 Solid-phase peptide synthesis

Full details of peptide sequences and final characterization can be found in Supplementary Table S2 and Figures S25–30. All peptides were synthesized on solid support using a Fmoc-based strategy (1). For manual peptide synthesis, a NovaSyn TGR® resin (*Merck*) solid support was used while H-Rink amide ChemMatrix® resin (*Sigma Aldrich*) for automated synthesis. General reagents and Fmoc-protected amino acids were purchased from *Avantor Performance Materials LLC*, *Carl Roth*, *Iris Biotech*, *Merck*, *Sigma Aldrich* and *Thermo Fisher Scientific*. (*R*)-*N*-Fmoc-2-(7'-octenyl)-alanine-OH (Fmoc-R_8-OH) and (*S*)-*N*-Fmoc-2-(4'-pentenyl)alanine-OH (Fmoc-S_5-OH) were purchased from *Okeanos Technology*.

Peptides **bt-B3**, **bt-C1**, **bt-D1**, **f-A3**, **f-B**, **f-B1**, **f-B2**, **f-B3**, **f-C**, **f-C1**, **f-C2**, **f-C3, f-wt33**, **f-wt36**, **f-wt41**, and **wt33** were synthesized manually in syringe reactors and mixing was performed using an orbital shaker. Between reaction steps, resins were washed with NMP (3x, 1 mL per 50 mg of resin), DCM (3x, 1 mL per 50 mg of resin), and NMP (3x, 1 mL per 50 mg of resin). To couple proteinogenic amino acids a solution containing 4 equivalents (eq.) of amino acid, 4 eq. COMU, and 4 eq. OXYMA in NMP (c = 0.25 M) was prepared and mixed with 8 eq. of DIPEA for 30 seconds before addition to the resin. After 40 min, the solution was discarded. A second coupling solution composed of 4 eq. amino acid and 4 eq. PyBOP in NMP (c = 0.25 M) with 8 eq. of NMM was added to the resin and incubated for 1 h. For the coupling of Fmoc-R_8-OH and Fmoc-S_5-OH, a solution of 3 eq. of building block, 3 eq. of PyBOP, and 6 eq. of DIPEA in NMP (c = 0.25 M) was added to the resin and reacted overnight. Subsequent proteinogenic amino acids were coupled as previously described but with an additional COMU coupling step. After each amino acid and building block coupling cycle, resins were treated with a capping solution of Ac_2O/DIPEA/NMP (*v*/*v*/*v*, 1/1/12) for 10 min. Subsequently, Fmoc deprotection was carried out by incubating resins in a solution of Piperidine/NMP (*v*/*v*, 1/4) for 15 min before moving to the next amino acid coupling cycle.

Peptides **B1**, **B3**, **C1**, **D1**, **f-wt26**, **f-wt33Δ4**, **f-A1**, **f-A2**, and **f-D1** were synthesized automatically using a Syro II (*MultiSynTech*), following a similar protocol as described for manual synthesis. For an automated procedure DMF was used as a wash solvent instead of NMP and all amino acids and building blocks were

dissolved with OXYMA in DMF (*c* = 0.5 M). Fmoc deprotection was performed in Piperidine/DMF (*v*/*v*, 2/5) for 3 min and subsequently again in Piperidine/DMF (*v*/*v*, 1/5) for 5 min. Amino acid and building block couplings were performed as triple couplings, incubating 4 eq. of amino acid or building block, 3.9 eq. PyBOP, and 8 eq. of DIPEA in DMF (*c* = 0.33 M) for 40 min for the first and third couplings, while for the second coupling HCTU was used as the coupling reagent. A 2 min capping step was performed at the end of each coupling cycle using Ac_2O/NMP (*v*/*v*, 1/10) and a solution of DIPEA/NMP (*c* = 1.33 M).

Peptides **B3a, B3b, B3c**, **B3d**, and **B3e** were synthesized automatically using a Syro I (*Multisyntech*). Fmoc-protected amino acids were prepared as 0.33 M solutions dissolved in DMF with 0.33 M OXYMA. Coupling reagents were dissolved in DMF (*c* = 0.33 M). DIPEA was dissolved in NMP (*c* = 1.33 M). Fmoc deprotection was carried out in piperidine/DMF (1/5, v/v), 2 x 5 min. Coupling was performed twice, Fmoc-Xaa-OH (4 eq.), PyBOP/HATU (3.9 eq.), and DIPEA (*c* = 1.33 M) for 30 min each. After double coupling, resins were treated with Ac_2O/DIPEA/NMP (1/1/10, v/v), 2 x 5 min.

Upon completion of the core amino acid sequence, the resins of peptides to be stapled were swollen in dry DCE for 1 h. A solution of Grubbs 1st generation catalyst (4 mg·mL^{-1}) in DCE was then added to the resin for 1 h while a continuous stream of nitrogen was bubbled through the reaction mixture. This procedure was repeated four more times with the resin being washed with DCE intermittently. Upon completion, the resin was washed with DMSO/DCM (*v*/*v*, 1/1) for 10 min. After Fmoc deprotection, final *N*-terminal modification was conducted. For *N*-terminal acetylation, resins were treated with a solution of Ac_2O/DIPEA/NMP (*v*/*v*/*v*, 1/1/12) for 10 min. *N*-terminal biotinylation was carried out by first coupling fluoro-enylmethyloxycarbonylamino-21-amino-4,7,10,13,16,19-hexaoxaheneicosanoic acid (Fmoc-Peg_5-OH) using the manual protocol for proteinogenic amino acids, followed by Fmoc deprotection, and finally the coupling of biotin using the same method. Fluorescent *N*-terminal modification was carried out by first coupling 2-[2-(fluoro-enylmethyloxycarbonylamino)ethoxy] acetic acid (Fmoc-Peg_2-OH) using the manual protocol for proteinogenic amino acids. After Fmoc deprotection, 4 eq. of FITC and 8 eq. of DIPEA in NMP (*c* = 0.25 M) were added to the resin. After 2 h, the solution was discarded and the reaction was repeated overnight.

After final *N*-terminal modification, resins were dried under vacuum and treated with a cleavage solution (TIPS/H_2O/EDT/TFA, *v*/*v*/*v*/*v*, 1/2.5/2.5/94; 1 mL per 50 mg of resin) at RT for 4 h. The resin was subsequently washed three times with additional cleavage solution. These solutions were combined and evaporated under a stream of nitrogen before the addition of cold diethyl ether to precipitate the peptide. After centrifugation (10 min, 4000 rpm, 4 °C) the supernatant was removed. Precipitated peptides were re-dissolved in a solution of ACN/H_2O (*v*/*v*, 3/10) for purification by reversed-phase HPLC (*Macherey-Nagel* Nucleodur C18 column;10×125 mm, 110 Å, 5 µm particle size, flow rate: 6 mL·min^{-1} or *Macherey-Nagel* Nucleodur C18 column; 21×125 mm, 110 Å, 5 µm particle size, flow rate: 17.5 mL·min^{-1}, A: water with 0.1 % TFA, B: ACN with 0.1 % TFA). A gradient elution was used with 0–50 % of solvent B over 40 min. Obtained pure fractions were pooled and lyophilized.

Peptide characterization was performed by analytical HPLC (1200 Series, *Agilent Technology*; flow rate: 1 mL·min^{-1}, A: water with 0.1 % TFA, B: ACN with 0.1 % TFA, Column: *Zorbax* Eclipse XDB-C18-column, 4.6×150 mm, 80 Å, 5 µm particle size) coupled with a mass spectrometer (6120 Quadrupole LC/MS, *Agilent Technology*). Analytical HPLC chromatograms recorded at λ = 210 nm and corresponding *m/z* ratios are shown in Supplementary Figures S25-S30. To reduce TFA salts, a solution of FA/ACN/H_2O (1/30/70, *v*/*v*/*v*) was added to the dried purified peptide and subsequently lyophilized overnight. The concentration of fluorescein-labeled peptides was determined by UV absorption in 100 mM sodium dihydrogen phosphate buffer (pH 8.5) at (λ = 494 nm, ε = 77,000 cm^{-1}·M^{-1}). Similarly, the concentration of *N*-terminally acetylated peptides containing tryptophan was determined by UV absorption at λ = 280 nm. Quantification of peptides **Bt-D1** and **D1** was performed by HPLC-based comparison (chromatogram, λ = 210 nm) with a gravimetrically-quantified reference peptide.

2. Supplementary Tables

Table S1: Overview of all oligonucleotides with corresponding sequence (from 5'-end to 3'-end, left to right), number of nucleotides (nt), and molecular weight (MW in g/mol). * = 5' phosphate.

Oligonucleotide	Sequence (5' – 3')	nt	MW (g/mol)
**pal-RNA**	*AGA CAG CAU UAU GCU GUC UUU	21	6715
**pal-DNA**	*AGA CAG CAT TAT GCT GTC TTT	21	6491
**miR-21 5'**	*UAG CUU AUC AGA CUG AUG UUG A	22	7084
**miR-21 3'**	*AAC ACC AGU CGA UGG GCU GU	21	6791
**pre-miR-21**	*UAG CUU AUC AGA CUG AUG UUG ACU GUU GAA UCU CAU GGC AAC ACC AGU CGA UGG GCU GU	59	18978
**HP 1**	GCU UAU CAG ACU GAU GUU GGA UCA ACA ACA UCA GUC UGA UAA GC	44	14075
**HP 2**	UAU UGU AUU AGU ACG CGC GGA UCA ACG CGC GUA CUA AUA CAA UA	44	14075
**HP 3**	GAA GUG UGC UCC UGU AUU AGA UCA AUA AUA CAG GAG CAC ACU UC	44	14075
**HP 4**	AUG UGU GCA UUA GAU CGU CGA UCA AGA CGA UCU AAU GCA CAC AU	44	14075
**HP 5**	GUG UUA CGG AUA UCC UGU AGA UCA AUA CAG GAU AUC CGU AAC AC	44	14075

Table S2: List of peptides with *N*-terminal modification, sequence (from *N*- to *C*-terminus), and calculated as well as found masses (m/z) for charged ions ($[M+nH]^{n+}$). Amino acids in one-letter code. f = fluorescein, Peg$_2$ = 8-amino-3,6-dioxaoctanoyl, Peg$_5$ = amino-penta(ethylene glycol)-omega-carboxyl, Ac = acetyl, Bt = *D*-biotin, J = 2-aminohexanoic acid, S_5: (*S*)-2-(4-pentenyl)alanine, and R_8: (*R*)-2-(7-octenyl)alanine.

Peptide	*N*-Term.	Sequence (*N* – *C*)	m/z calc.	m/z found
**wt26**	f-Peg$_2$	RHKLNRKERGHKSPSEQRRSELWHAR	954.3	954.3 $[M+4H]^{4+}$
**wt33**	f-Peg$_2$<br>Ac	KKQAQRKRHKLNRKERGHKSPSEQRRSELWHAR	1171.3<br>1048.2	1171.5 $[M+H]^{4+}$<br>1048.1 $[M+4H]^{4+}$
**wt33Δ4**	f-Peg$_2$	KKQAQRKRHKLNRKERGHKSPSEQRRSEL	1033.7	1033.8 $[M+4H]^{4+}$
**wt36**	f-Peg$_2$	JNQKKQAQRKRHKLNRKERGHKSPSEQRRSELWHAR	1260.2	1260.2 $[M+4H]^{4+}$
**wt41**	f-Peg$_2$	RKLERJNQKKQAQRKRHKLNRKERGHKSPSEQRRSELWHAR	1430.9	1431.0 $[M+4H]^{4+}$
**A1**	f-Peg$_2$	KKQR_8QRKRHKS_5NRKERGHKSPSEQRRSELWHAR	1198.4	1198.4 $[M+4H]^{4+}$
**A2**	f-Peg$_2$	KKQAQRS_5RHKS_5NRKERGHKSPSEQRRSELWHAR	1173.6	1173.5 $[M+4H]^{4+}$
**A3**	f-Peg$_2$	KKQAQRKRHKS_5NRKS_5RGHKSPSEQRRSELWHAR	1173.4	1173.2 $[M+4H]^{4+}$
**B**	f-Peg$_2$	KKQAQRKRHKLNRKERGHKSPSES_5RRSS_5LWHAR	1169.9	1169.8 $[M+4H]^{4+}$
**B1**	f-Peg$_2$<br>Ac	KKQR_8QRKRHKS_5NRKERGHKSPSES_5RRSS_5LWHAR	1196.7<br>1073.5	1196.3 $[M+4H]^{4+}$<br>1073.7 $[M+4H]^{4+}$
**B2**	f-Peg$_2$	KKQAQRS_5RHKS_5NRKERGHKSPSES_5RRSS_5LWHAR	1172.1	1171.8 $[M+4H]^{4+}$
**B3**	f-Peg$_2$<br>Ac<br>Bt-Peg$_5$	KKQAQRKRHKS_5NRKS_5RGHKSPSES_5RRSS_5LWHAR	1171.9<br>1048.5<br>1167.4	1171.6 $[M+4H]^{4+}$<br>1048.4 $[M+4H]^{4+}$<br>1167.4 $[M+4H]^{4+}$
**B3a**	Ac	KKQAQRKRHKS_5NRKS_5RGHKSPSES_5RESS_5LWHAR	1041.7	1041.6 $[M+4H]^{4+}$
**B3b**	Ac	KKQAQRKRHKS_5NEKS_5RGHKSPSES_5RRSS_5LWHAR	833.6	833.7 $[M+5H]^{5+}$
**B3c**	Ac	KKQAQRKRHKS_5NEKS_5RGHKSPSES_5RESS_5LWHAR	1034.9	1034.6 $[M+4H]^{4+}$
**B3d**	Ac	KKQAQEKRHKS_5NRKS_5RGHKSPSES_5RESS_5LWHAR	1034.9	1034.7 $[M+4H]^{4+}$
**B3e**	Ac	KKQAQEKRHKS_5NEKS_5RGHKSPSES_5RESS_5LWHAR	1028.2	1027.9 $[M+4H]^{4+}$
**C**	f-Peg$_2$	KKQAQRKRHKLNRKERGHKSPSEQRRSS_5LWHS_5R	1183.9	1183.9 $[M+4H]^{4+}$
**C1**	f-Peg$_2$<br>Ac<br>Bt-Peg$_5$	KKQR_8QRKRHKS_5NRKERGHKSPSEQRRSS_5LWHS_5R	1210.9<br>1087.8<br>1206.7	1210.9 $[M+4H]^{4+}$<br>1087.8 $[M+4H]^{4+}$<br>1206.7 $[M+4H]^{4+}$
**C2**	f-Peg$_2$	KKQAQRS_5RHKS_5NRKERGHKSPSEQRRSS_5LWHS_5R	1186.1	1185.9 $[M+4H]^{4+}$
**C3**	f-Peg$_2$	KKQAQRKRHKS_5NRKS_5RGHKSPSEQRRSS_5LWHS_5R	1185.9	1185.7 $[M+4H]^{4+}$
**D1**	f-Peg$_2$<br>Ac<br>Bt-Peg$_5$	KKQAQRKRHR_8LNRKERS_5HKSPSEQRS_5SELS_5HAR	1175.1<br>1052.0<br>1170.7	1175.3 $[M+4H]^{4+}$<br>1052.0 $[M+4H]^{4+}$<br>1170.9 $[M+4H]^{4+}$

Table S3: Overview of isothermal titration calorimetry data for *N*-terminally acetylated peptides **wt33**, **B1**, **B3**, and **C1** binding to double-stranded palindromic RNA (pal-RNA), double-stranded microRNA-21 (miR-21), and pre-miR-21. Measurements were performed in triplicate.

Peptide	RNA	K_d / µM	N	ΔH / kcal·mol^{-1}	$-T\Delta \cdot S$ / kcal·mol^{-1}	ΔG / kcal·mol^{-1}
**wt33**	pal-RNA	1.19 ±0.02	0.47 ±0.01	-50.1 ±0.3	41.8 ±0.3	-8.3 ±0.1
**B1**	pal-RNA	0.26 ±0.01	0.47 ±0.02	-39.3 ±0.2	30.2 ±0.2	-9.1 ±0.02
**B3**	pal-RNA	0.07 ±0.01	0.62 ±0.02	-40.4 ±0.1	30.4 ±0.1	-9.9 ±0.01
	miR-21	0.018 ±0.002	0.43 ±0.01	-39.9 ±0.01	29.1 ±0.02	-10.7 ±0.01
	pre-miR-21	0.075 ±0.008	0.28 ±0.01	-62.1 ±1.6	52.1 ±1.7	-9.9 ±0.05
**C1**	pal-RNA	0.18 ±0.01	0.57 ±0.01	-41.0 ±0.5	31.6 ±0.5	-9.4 ±0.06

Table S4: Secondary structure distribution obtained from circular dichroism measurements with *N*-terminally fluorescein-labeled peptides calculated using CDNN/PEPFIT(2, 3). Percentages of calculated secondary structure species (helix, β-sheet, β-turn and random coil) are presented.

Peptide	Helix	β-Sheet	β-Turn	Random coil	Total Sum
**wt33**	7.3	5.3	4.2	83.7	100.5
**A1**	39.1	2.9	9.6	51.4	103.0
**A2**	21.9	21.6	7.9	51.3	100.7
**A3**	24.0	10.6	10.1	52.8	97.5
**B**	30.5	6.8	13.9	46.0	97.2
**B1**	81.8	19.0	13.1	13.8	110.6
**B2**	82.2	2.4	10.3	12.5	108.0
**B3**	85.0	2.0	13.9	9.3	110.2
**C**	28.4	2.8	7.6	63.6	102.4
**C1**	81.5	0.2	1.8	47.0	130.5
**C2**	61.8	3.3	4.2	41.6	110.9
**C3**	59.3	4.3	6.2	37.0	106.8
**D1**	48.5	12.0	9.9	31.2	101.6

Table S5: Half-life ($t_{1/2}$) values in min of *N*-terminally fluorescein-labeled peptides in protease stability assay.

Peptide	$t_{1/2}$ / min
**wt33**	2.4
**A1**	2.4
**A2**	2.3
**A3**	3.5
**B**	64.9
**B1**	829
**B2**	536
**B3**	371
**C**	77.7
**C1**	636
**C2**	440
**C3**	303
**D1**	279

3. Supplementary Figures

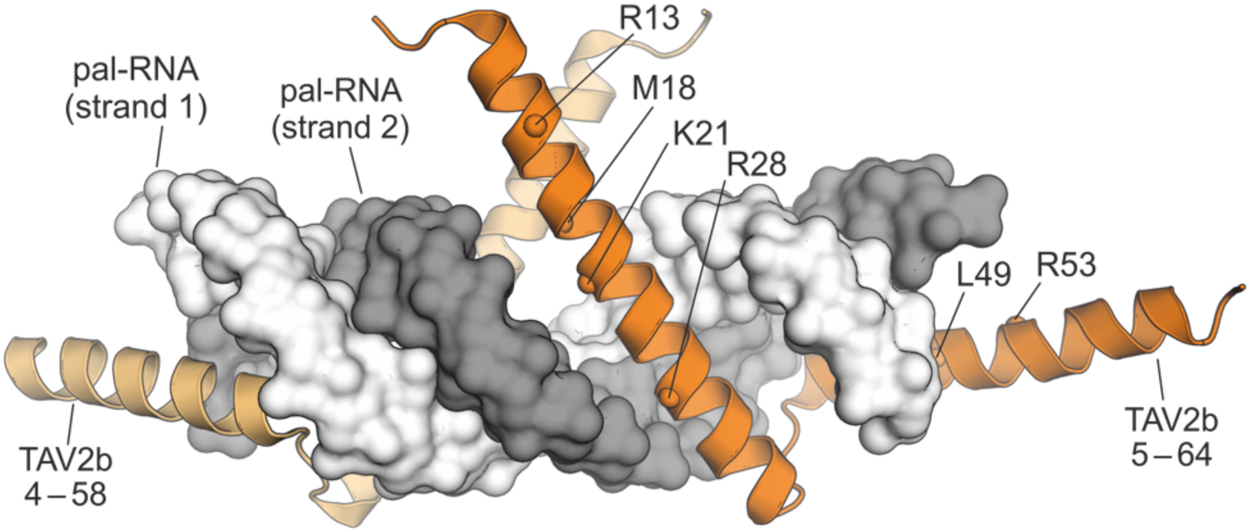

Figure S1: Crystal structure (PDB ID: 2ZI0) of two TAV2b units (I4-S58 and E5-N64, orange, cartoon representation) in complex with double-stranded palindromic RNA (pal-RNA, light/dark gray, single strand sequence: AGACAGCAUUAUGCUGUCUU). The pal-RNA sequence used for crystallization included an additional 3' uridine which was, however, not resolved in this crystal structure. Selected side chain positions for stapling are represented as spheres (4).

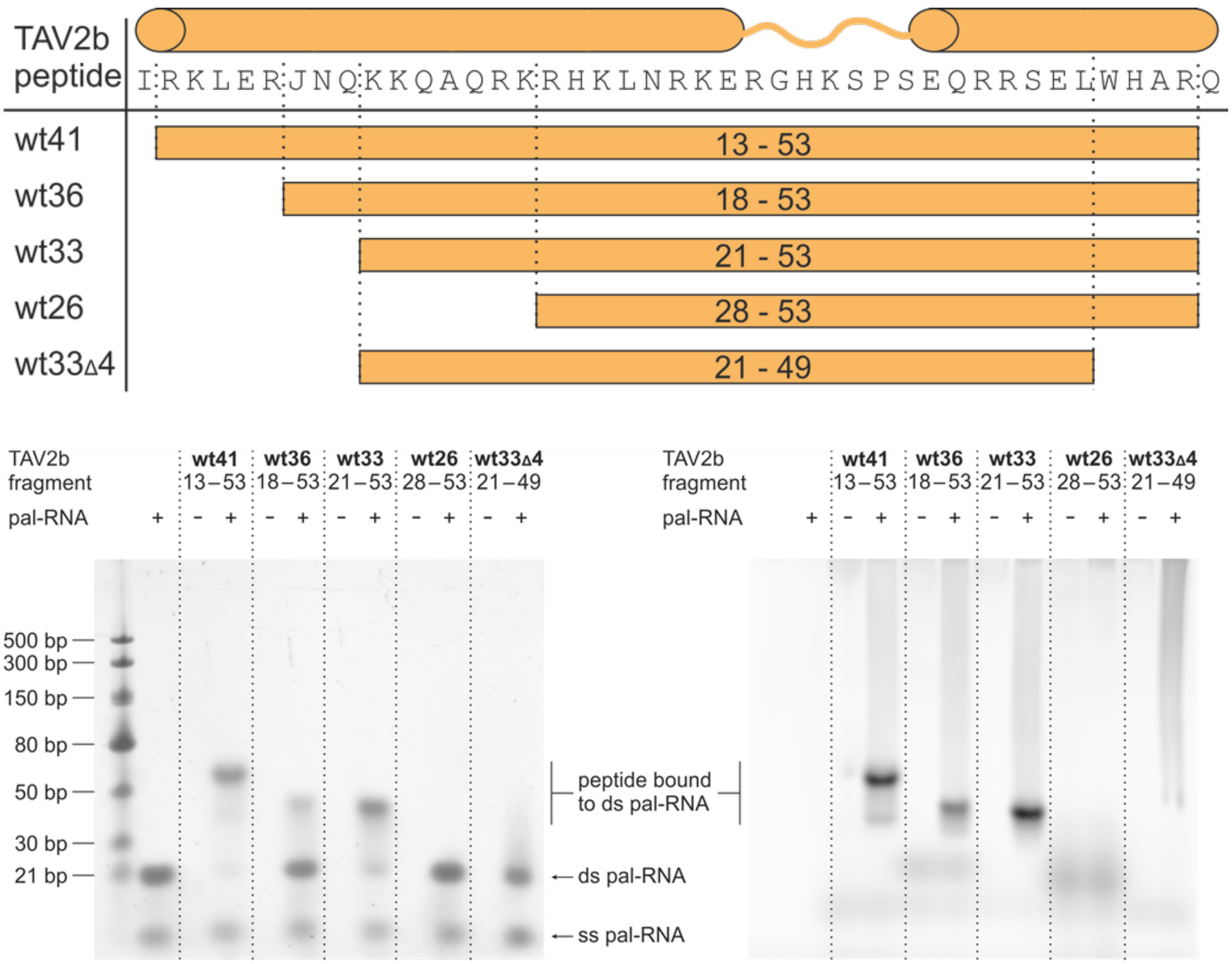

Figure S2: (*Top*) The sequences of TAV2b-derived peptides used in truncation experiments. (*Bottom*) EMSA of pal-RNA (c = 3 μM) incubated with *N*-terminally fluorescein-labeled peptides (c = 6 μM). *Left,* gel imaged after SYBR^{TM} gold staining. *Right,* gel imaged for fluorescein fluorescence. ds = double-stranded and ss = single-stranded.

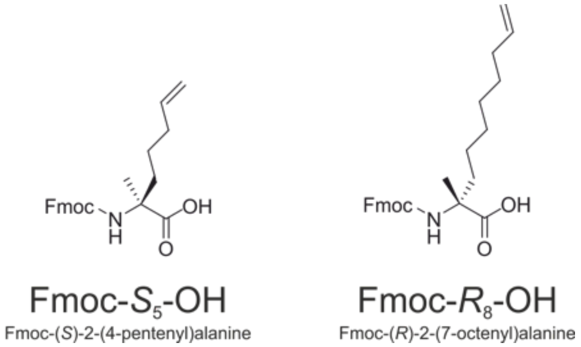

Figure S3: Fmoc-protected, non-natural amino acids as used in solid-phase peptide synthesis (Fmoc-S_5-OH: Fmoc-(*S*)-2-(4-pentenyl)-alanine; Fmoc-R_8-OH: Fmoc-(*R*)-2-(7-octenyl)alanine).

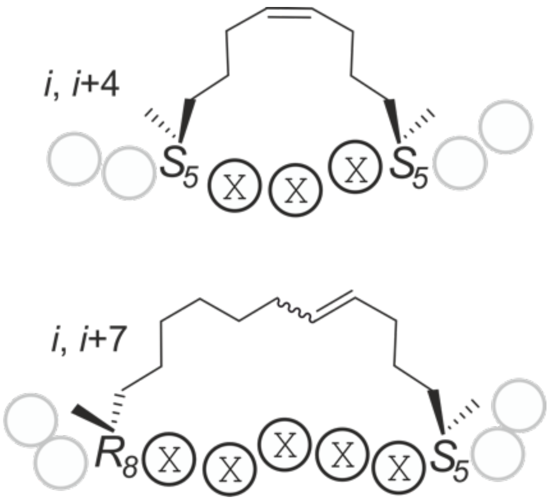

Figure S4: Chemical structures of *i*, *i*+4 and *i*, *i*+7 staple architectures (S_5 = (*S*)-2-(4-pentenyl)-alanine, R_8 = (*R*)-2-(7-octenyl)alanine).

wt33/pal-RNA measurement 1

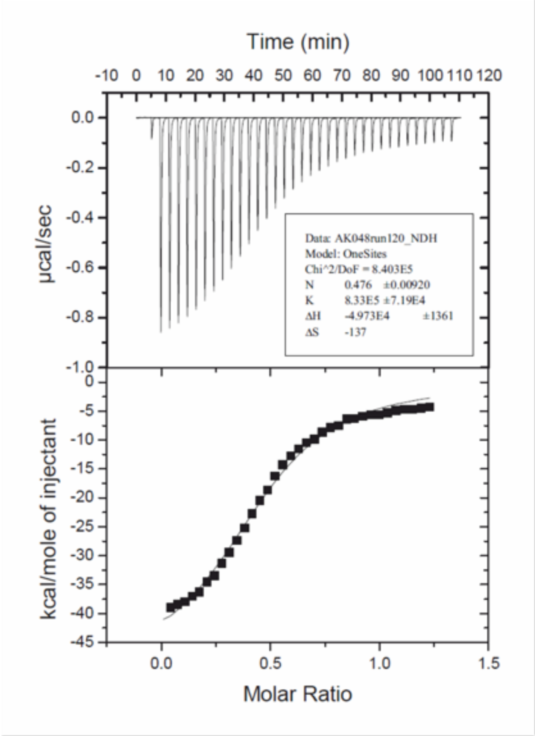

wt33/pal-RNA measurement 2

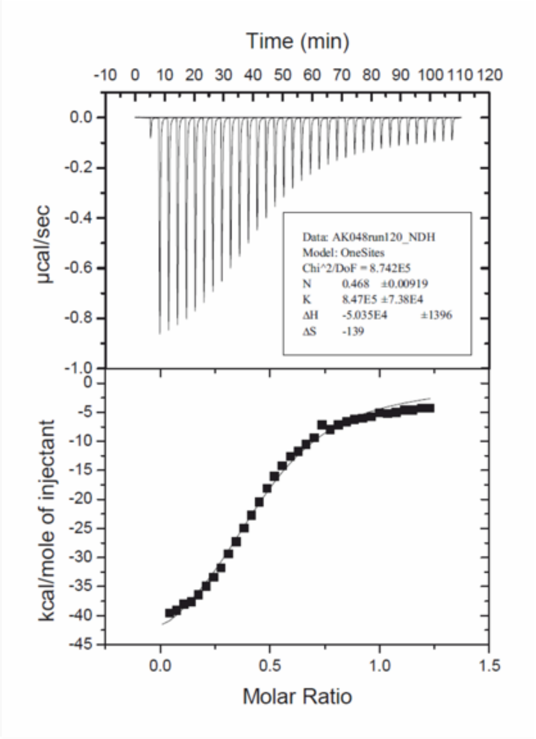

wt33/pal-RNA measurement 3

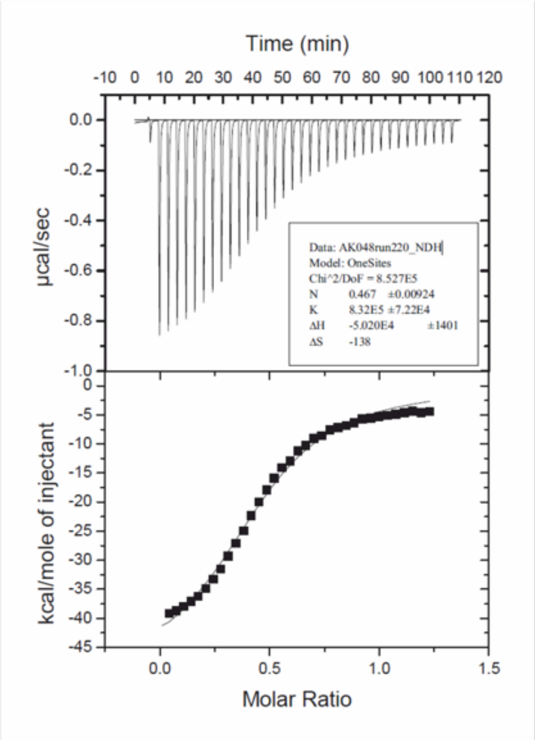

Figure S5: ITC measurements of *N*-terminally acetylated peptide **wt33** with pal-RNA. Measurements were performed in triplicate (*c*(peptide) = 12 µM and *c*(pal-RNA) = 72 µM).

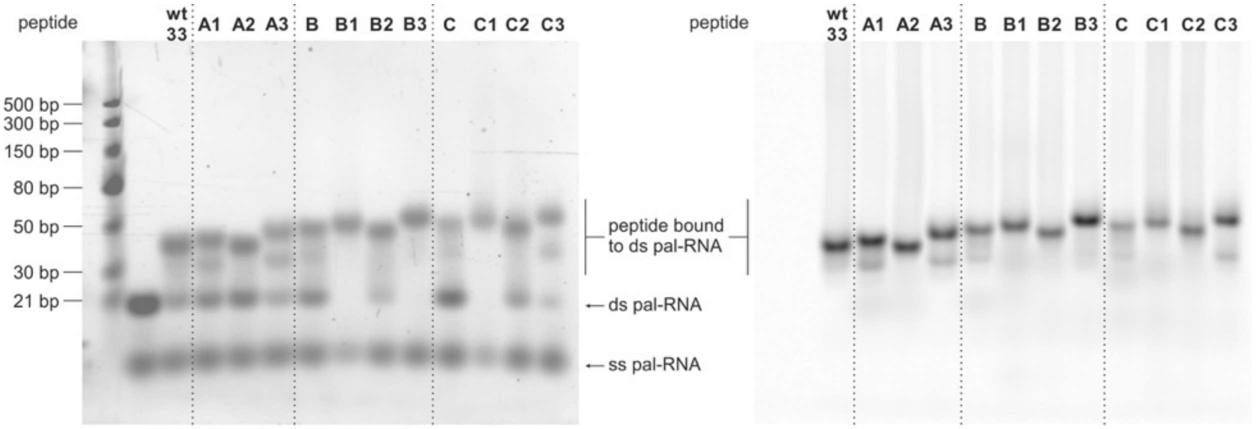

Figure S6: EMSA of pal-RNA (c = 3 μM) incubated with *N*-terminally fluorescein-labeled peptides (c = 6 μM). *Left,* gel imaged after SYBR™ gold staining. *Right,* gel imaged for fluorescein fluorescence (ds = double-stranded and ss = single-stranded).

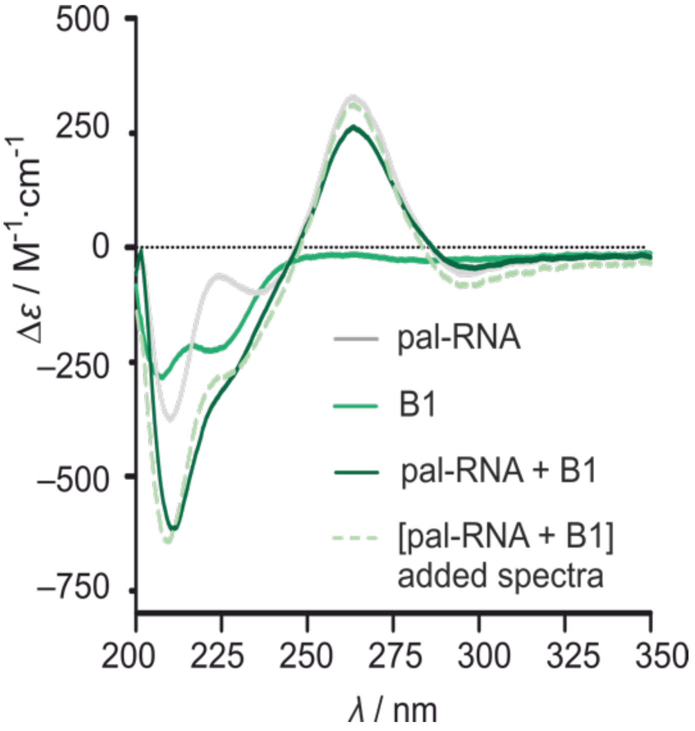

Figure S7: CD spectra of pal-RNA, *N*-terminally acetylated peptide **B1**, spectra of pal-RNA incubated with **B1**, and the sum of the two spectra ([pal-RNA + **B1**]).

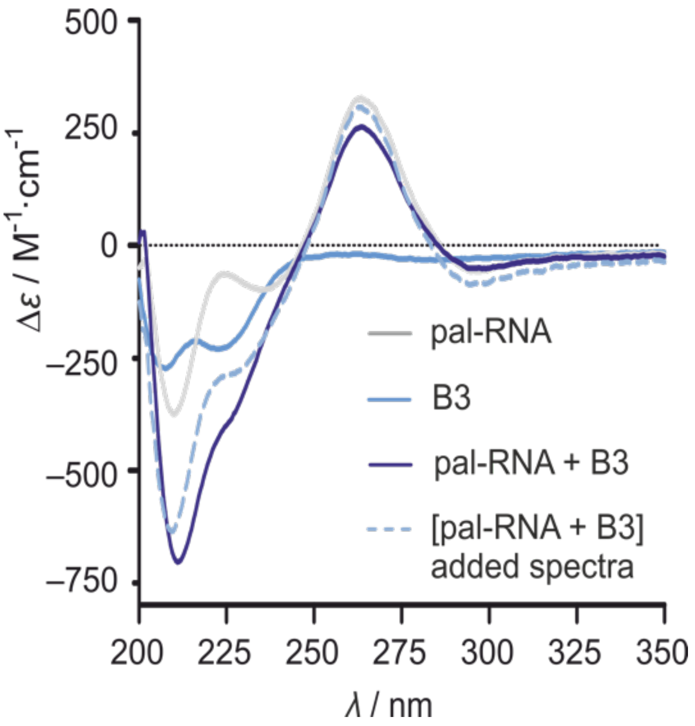

Figure S8: CD spectra of pal-RNA, *N*-terminally acetylated peptide **B3**, spectra of pal-RNA incubated with **B3**, and the sum of the two spectra ([pal-RNA + **B3**]).

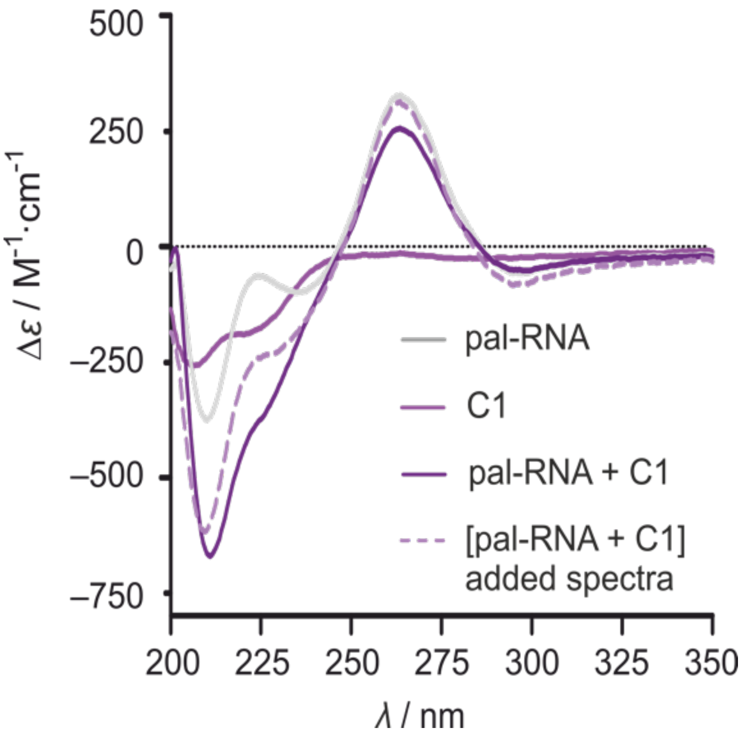

Figure S9: CD spectra of pal-RNA, *N*-terminally acetylated peptide **C1**, spectra of pal-RNA incubated with **C1**, and the sum of the two spectra ([pal-RNA + **C1**]).

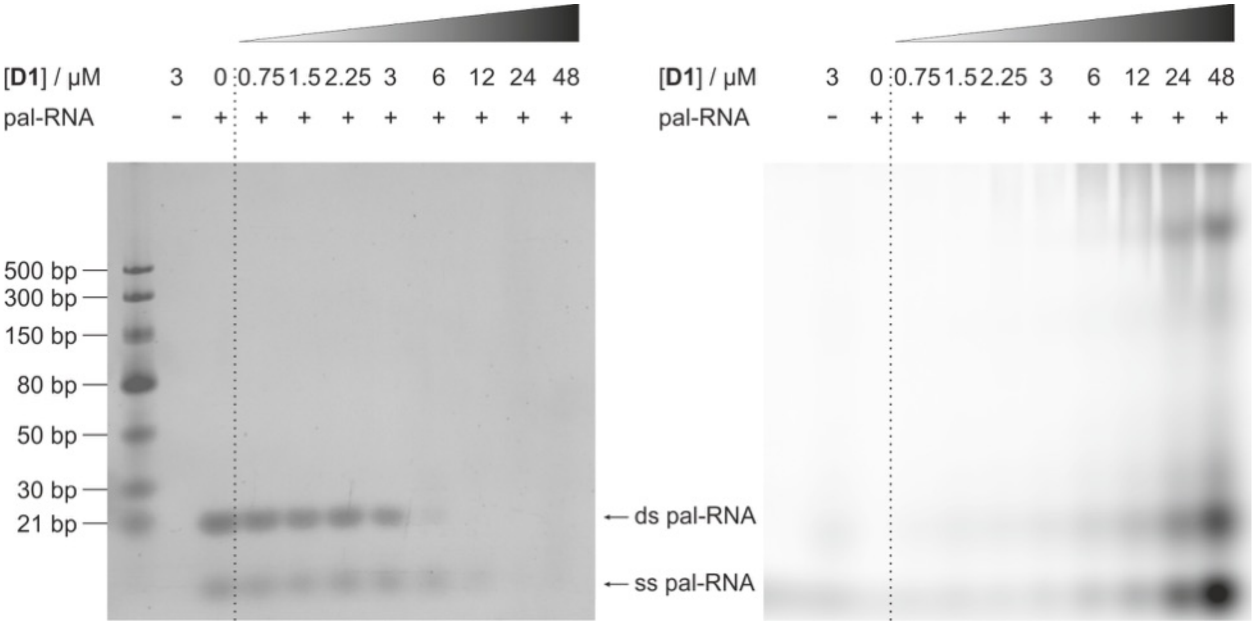

Figure S10: EMSA of pal-RNA (*c* = 1.5 µM) incubated with increasing concentrations of *N*-terminally fluorescein-labeled peptide **D1** (*c* = 0.75 - 48 µM). *Left,* gel imaged after SYBR^{TM} gold staining. *Right,* gel imaged for fluorescein fluorescence (ds = double-stranded and ss = single-stranded).

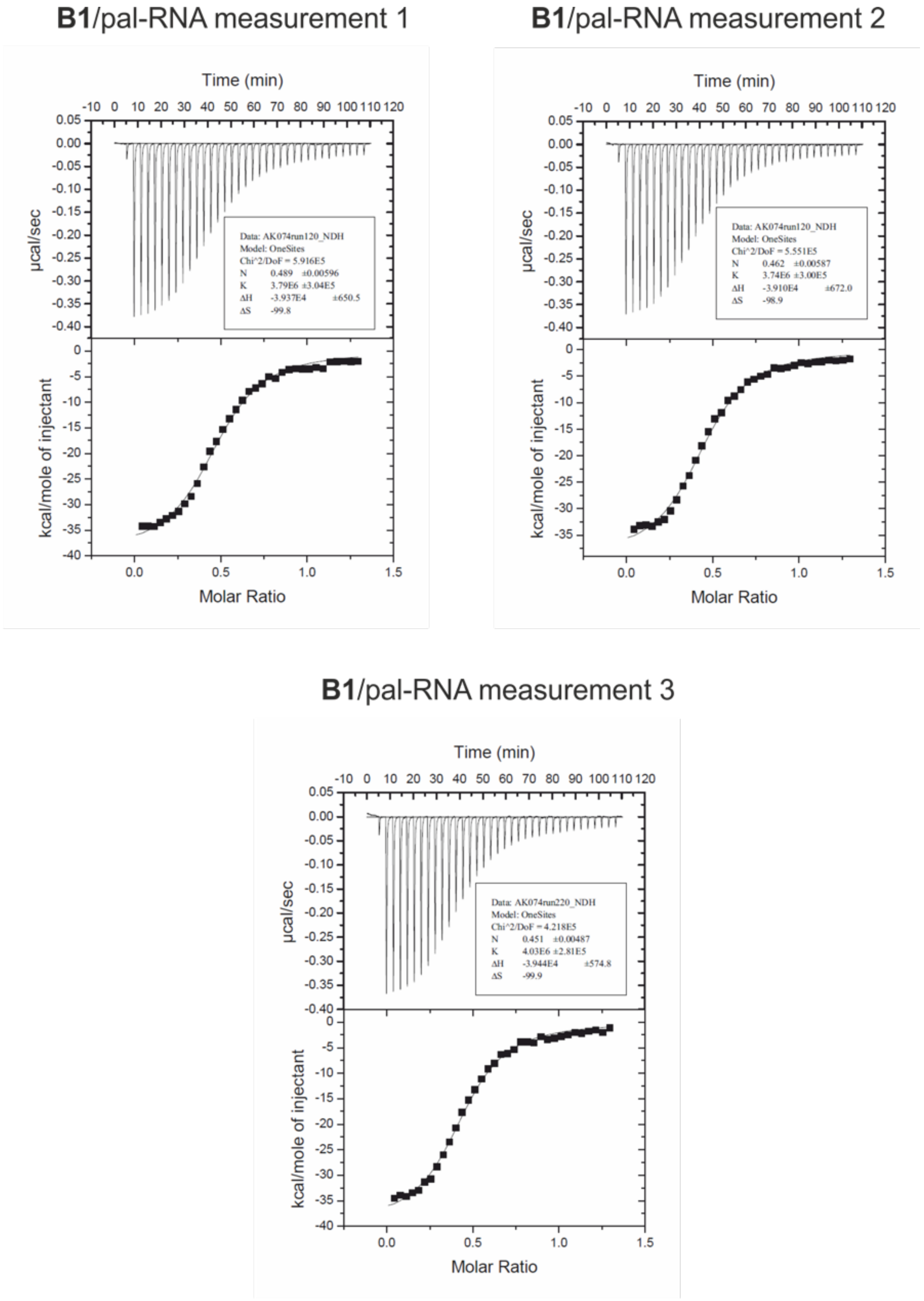

Figure S11: ITC measurements of *N*-terminally acetylated peptide **B1** with pal-RNA. Measurements were performed in triplicate (*c*(peptide) = 6 µM and *c*(pal-RNA) = 36 µM).

B3/pal-RNA measurement 1

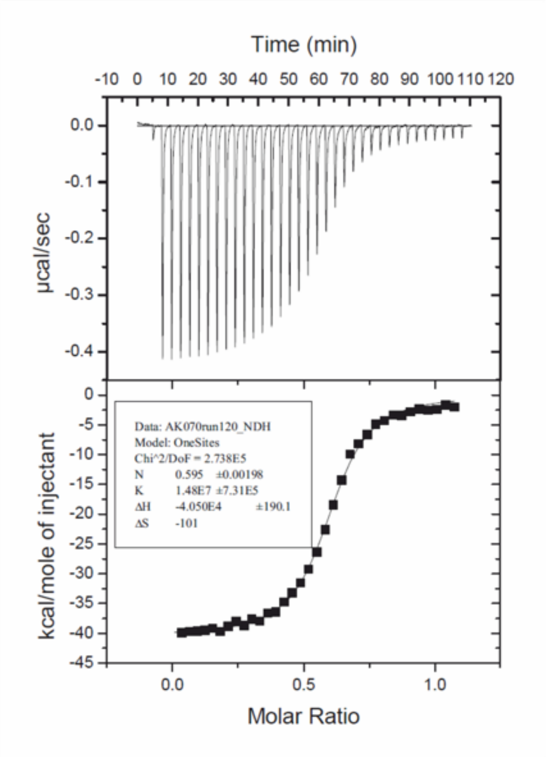

B3/pal-RNA measurement 2

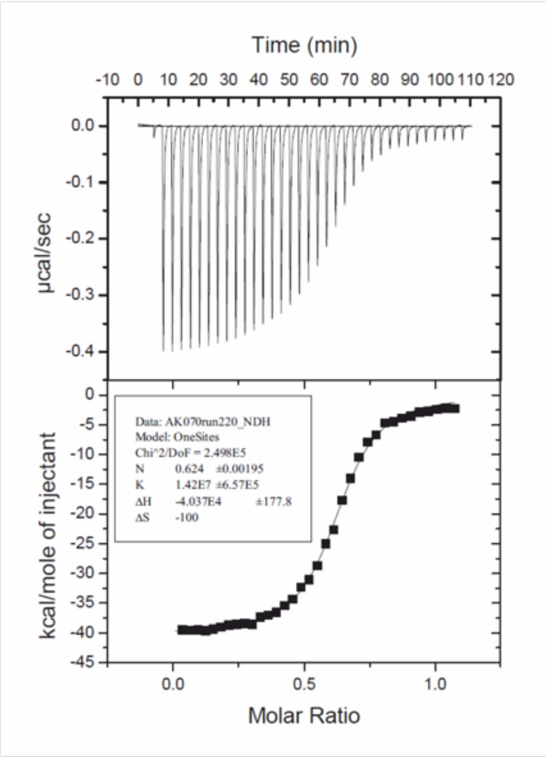

B3/pal-RNA measurement 3

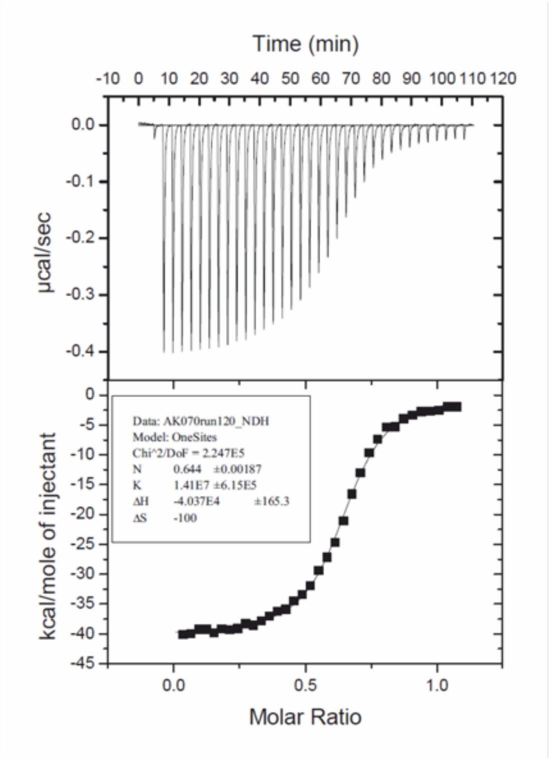

Figure S12: ITC measurements of *N*-terminally acetylated peptide **B3** with pal-RNA. Measurements were performed in triplicate (c(peptide) = 6 µM and c(pal-RNA) = 36 µM).

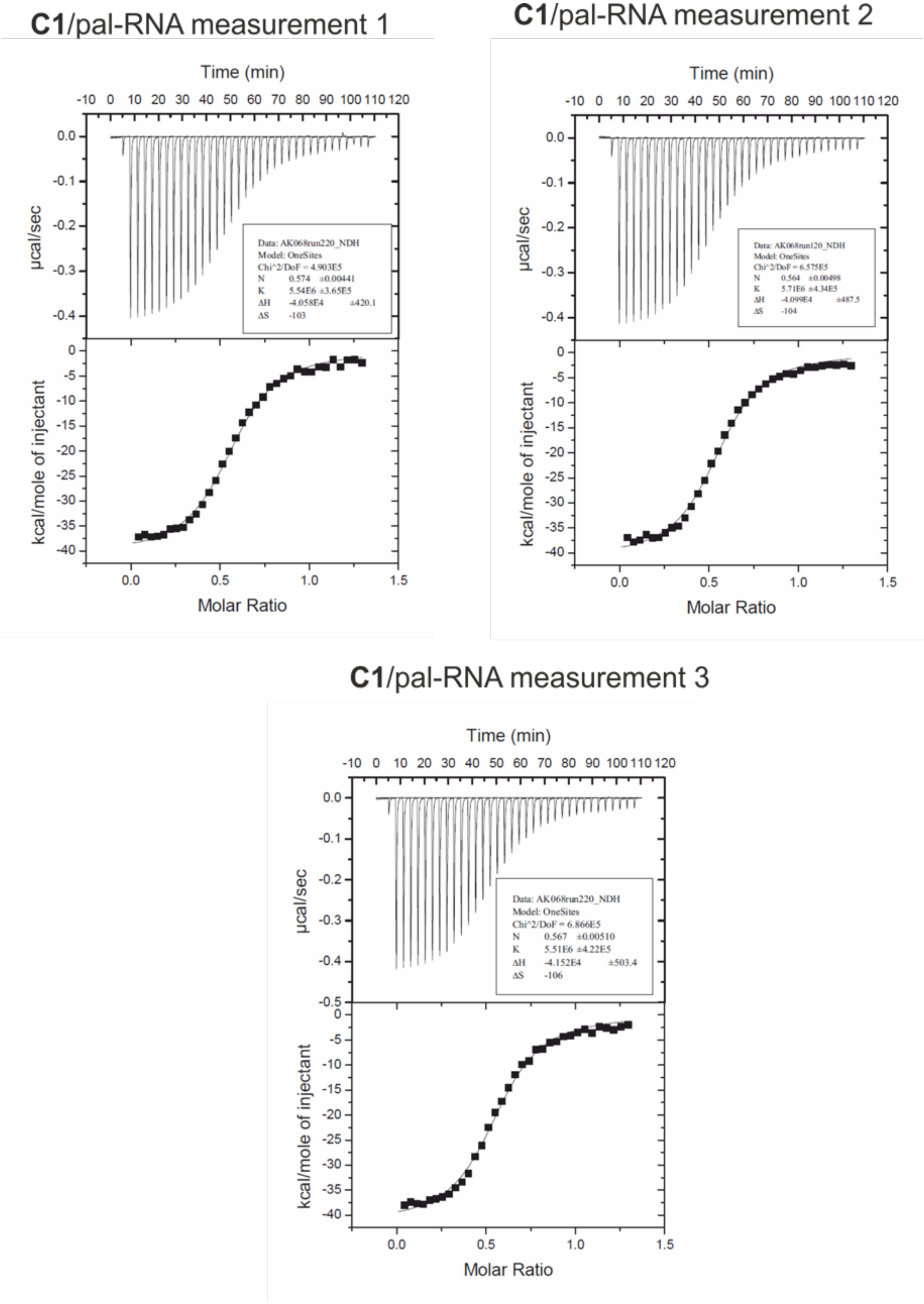

Figure S13: ITC measurements of *N*-terminally acetylated peptide **C1** with pal-RNA. Measurements were performed in triplicate (*c*(peptide) = 6 µM and *c*(pal-RNA) = 36 µM).

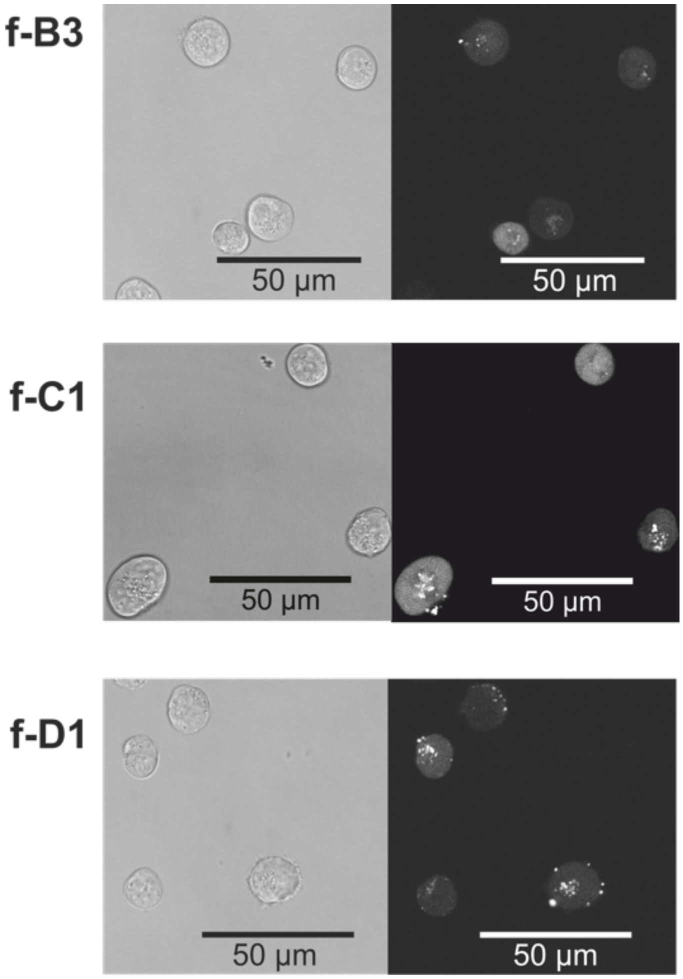

Figure S14: Live-cell confocal microscopy of K562 cells treated with fluorescently-labeled derivatives of peptides **B3** (*Top*), **C1** (*Middle*), and **D1** (*Bottom*) (c = 1 µM, t = 30 min).

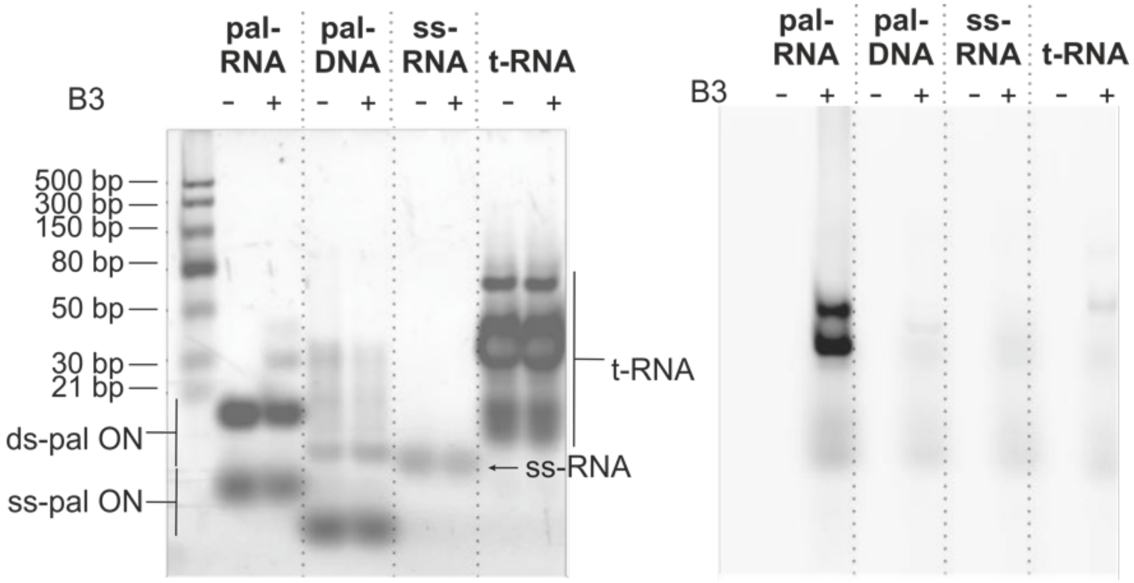

Figure S15: EMSA of *N*-terminally fluorescein-labeled peptide **B3** (*c* = 3 μM) incubated with pal-RNA, double-stranded palindromic DNA (pal-DNA), single stranded RNA (ss-RNA miR-21 3´), and transfer RNA (t-RNA) (*c* = 9 μM). *Left,* gel imaged after SYBR^{TM} gold staining. *Right,* gel imaged for fluorescein fluorescence (ds = double-stranded and ON = oligonucleotide).

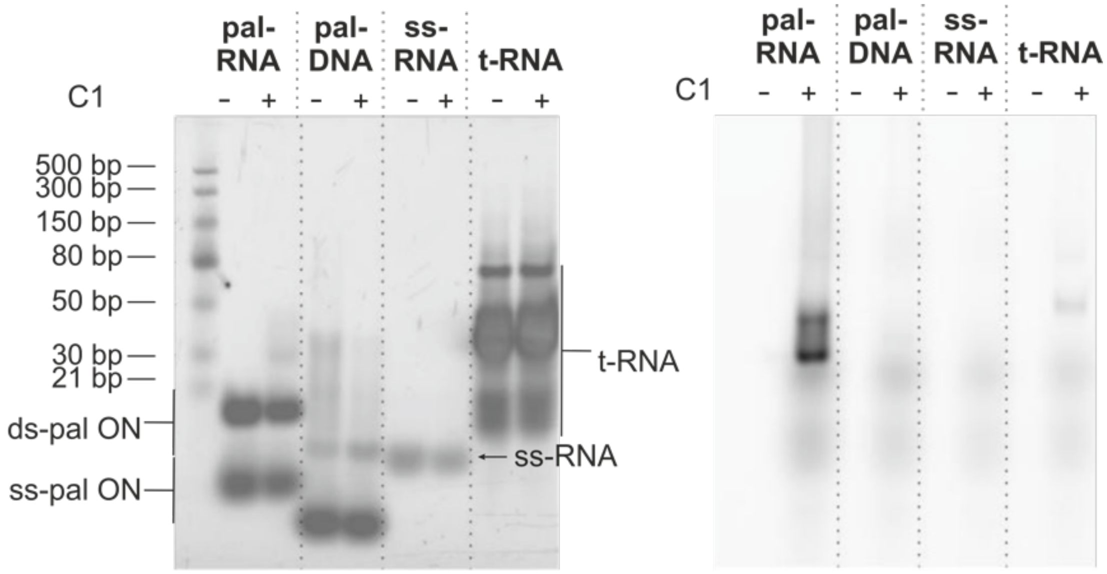

Figure S16: EMSA of *N*-terminally fluorescein-labeled peptide **C1** (*c* = 3 μM) incubated with pal-RNA, double-stranded palindromic DNA (pal-DNA), single stranded RNA (ss-RNA miR-21 3´), and transfer RNA (t-RNA) (*c* = 9 μM). *Left,* gel imaged after SYBR^{TM} gold staining. *Right,* gel imaged for fluorescein fluorescence (ds = double-stranded and ON = oligonucleotide).

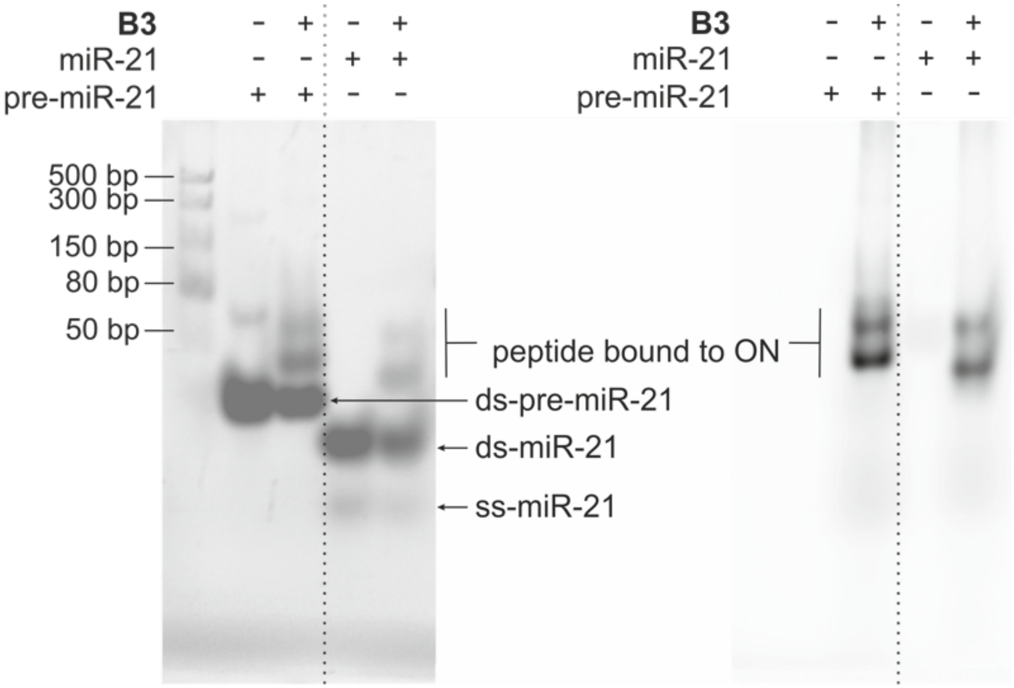

Figure S17: EMSA of ds miR-21 and pre-miR-21 (c = 3 μM) incubated with *N*-terminally fluorescein-labeled-peptide **B3** (c = 6 μM). *Left,* gel imaged after SYBR^{TM} gold staining. *Right,* gel imaged for fluorescein fluorescence (ON = oligonucleotide, ds = double-stranded, and ss = single-stranded).

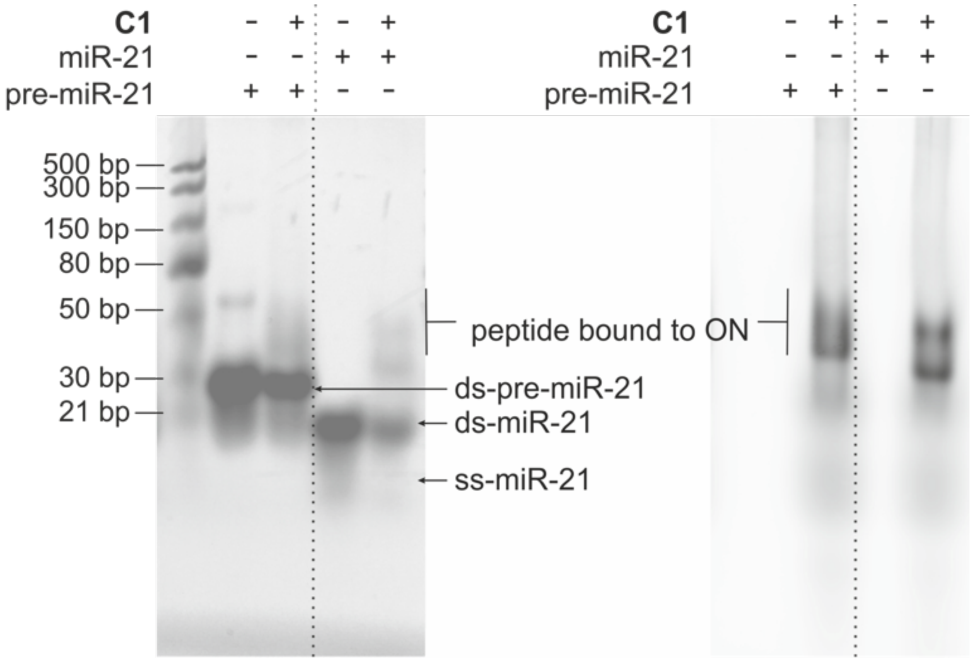

Figure S18: EMSA of ds miR-21 and pre-miR-21 (c = 3 μM) incubated with *N*-terminally fluorescein-labeled peptide **C1** (c = 6 μM). *Left,* gel imaged after SYBR^{TM} gold staining. *Right,* gel imaged for fluorescein fluorescence (ON = oligonucleotide, ds = double-stranded, and ss = single-stranded).

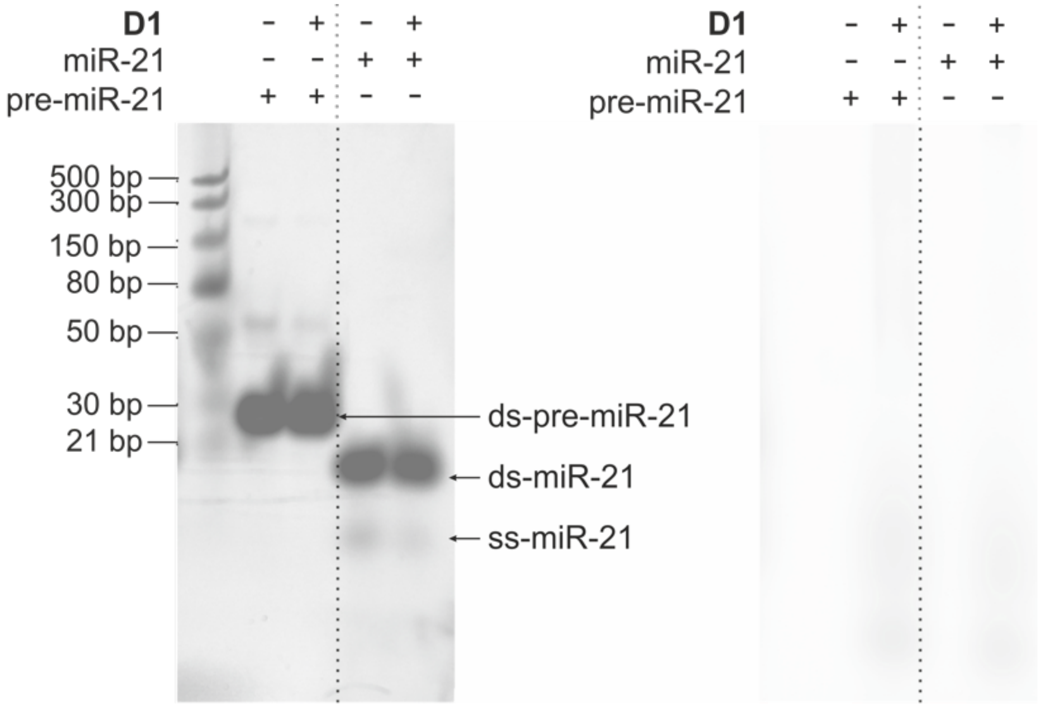

Figure S19: EMSA of ds miR-21 and pre-miR-21 (c = 3 μM) incubated with *N*-terminally fluorescein-labeled peptide **D1** (c = 6 μM). *Left,* gel imaged after SYBR^{TM} gold staining. *Right,* gel imaged for fluorescein fluorescence (ds = double-stranded and ss = single-stranded).

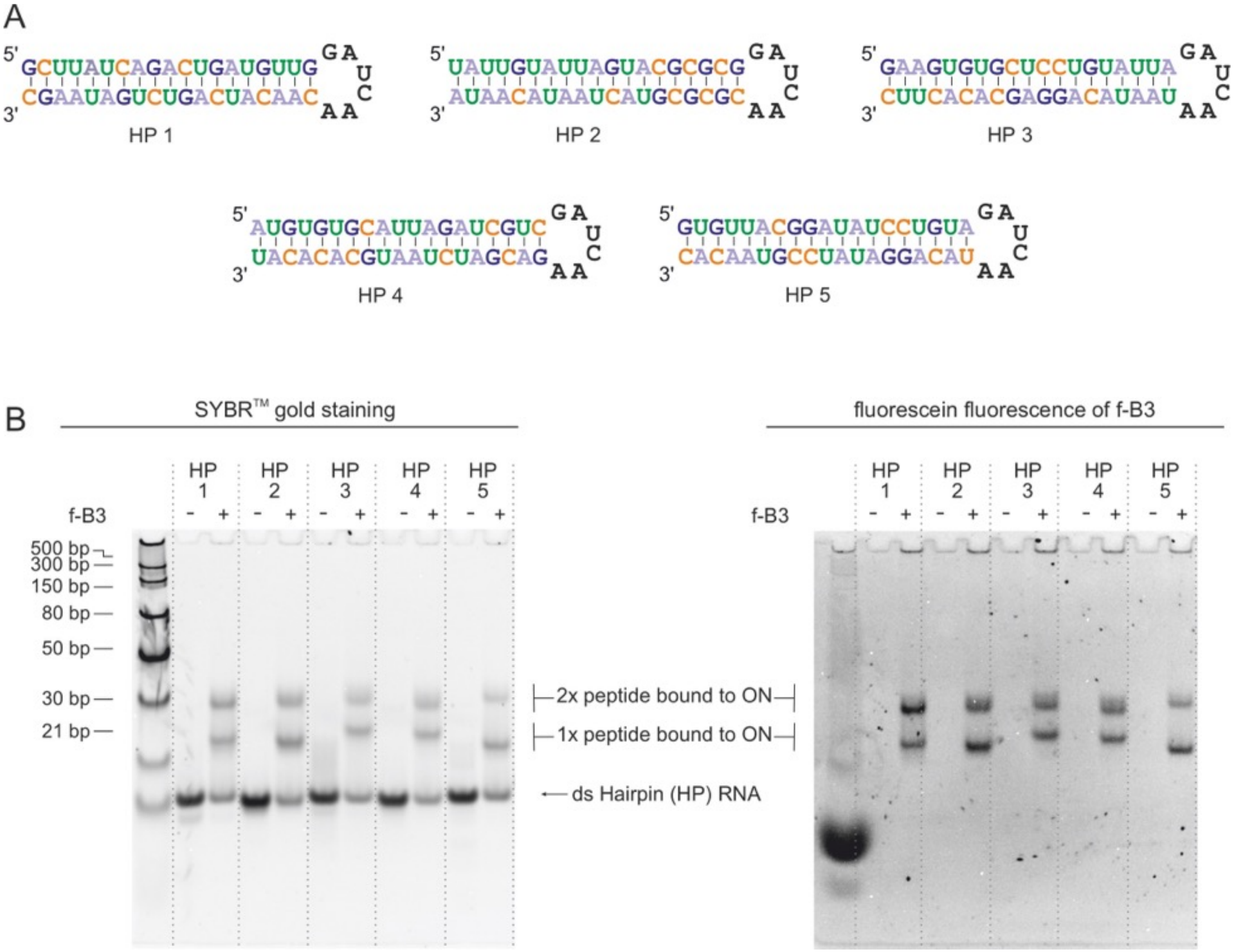

Figure S20: (A) Sequences of RNA hairpins (HP 1–5). All hairpins bear the same loop (black, GAUCAA). Stem of HP 1 has been derived from miR-21 while the other stems have been generated randomly. All sequences have been checked for alternative secondary structures to ensure hairpin formation. (B) EMSA of hairpin sequences (HP 1–5, c = 1 µM) incubated with **f-B3** (c = 2 µM). *Left,* gel imaged after SYBR™ gold staining. *Right,* gel imaged for fluorescein fluorescence (ON = oligonucleotide, ds = double-stranded, and ss = single-stranded).

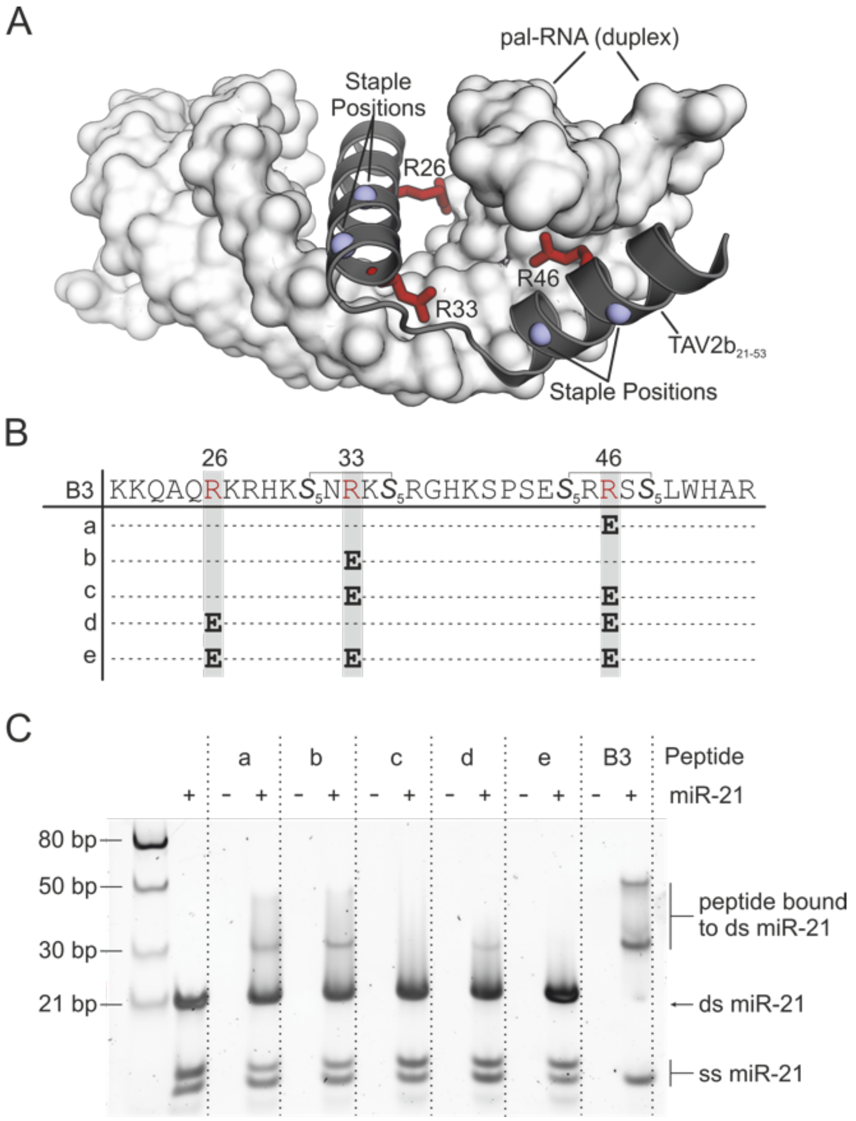

Figure S21: (A) Crystal structure of TAV2b (PDB ID: 2ZI0) showing only the **wt33** sequence (K21–R53, gray, cartoon representation) in complex with ds pal-RNA (white, surface representation). Selected arginine (R) side chains are shown in red. Positions used for the insertion of hydrocarbon staples are indicated with blue spheres. (B) Sequences of **B3**-derived peptide variants, highlighting positions of arginine to glutamic acid variation. (C) EMSA of ds miR-21 (c = 3 µM) incubated with **B3**-derived peptides (c = 6 µM), gel imaged after SYBR^{TM} gold staining (ds = double-stranded, and ss = single-stranded).

B3/miR-21 measurement 1

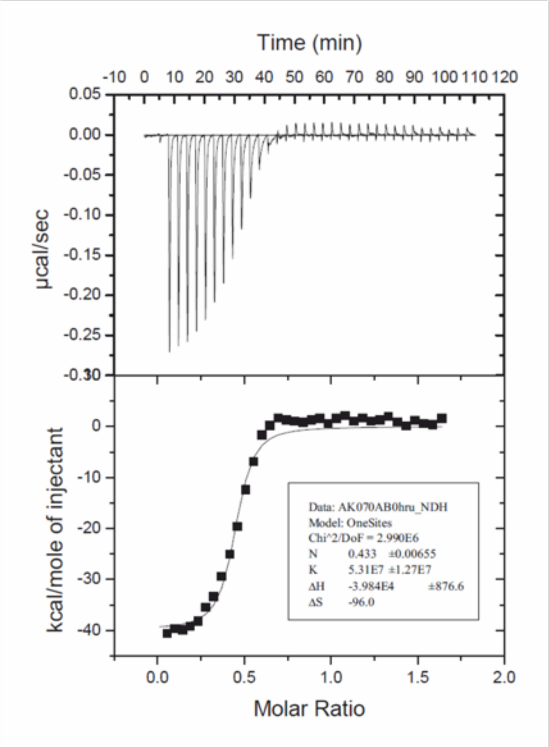

B3/miR-21 measurement 2

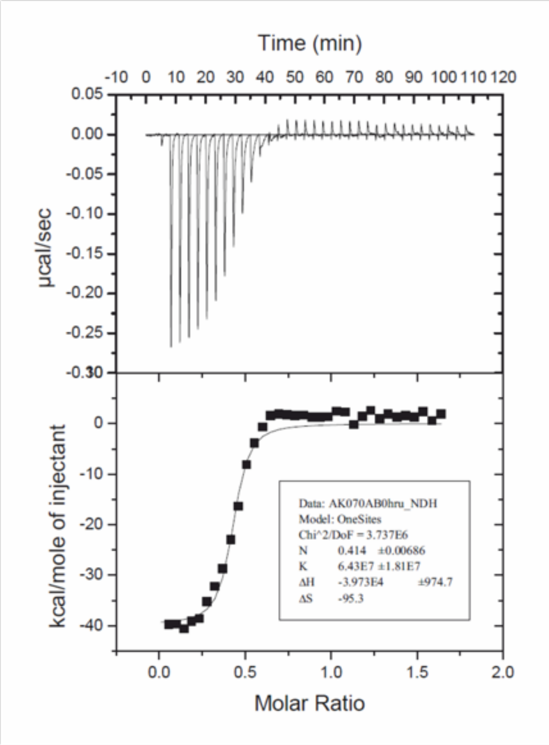

B3/miR-21 measurement 3

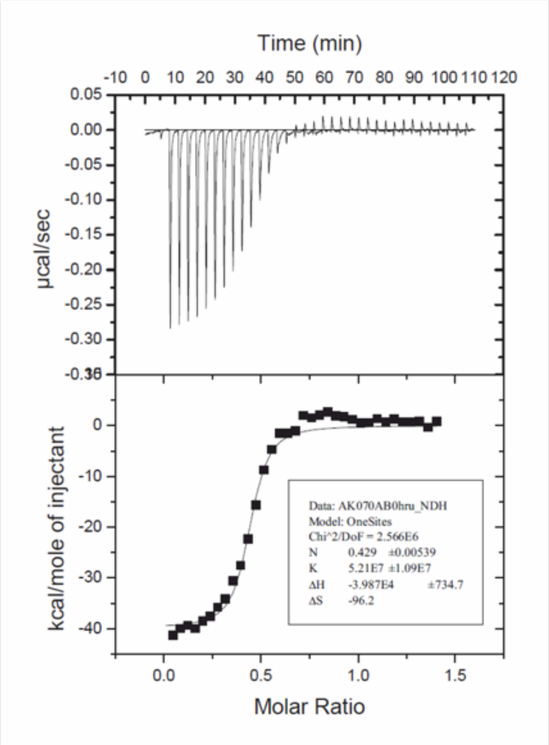

Figure S22: ITC measurements of *N*-terminally acetylated peptide **B3** with ds miR-21. Measurements were performed in triplicate (*c*(peptide) = 3 µM and *c*(miR-21-RNA) = 24 µM).

B3/pre-miR-21 measurement 1

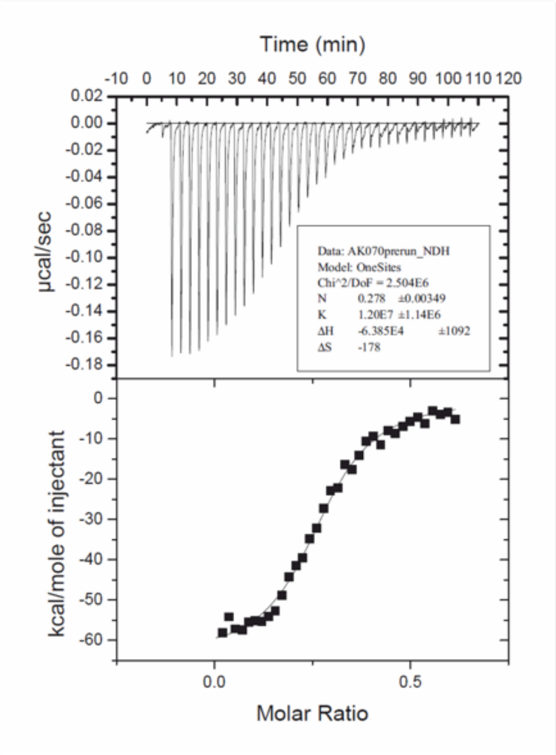

B3/pre-miR-21 measurement 2

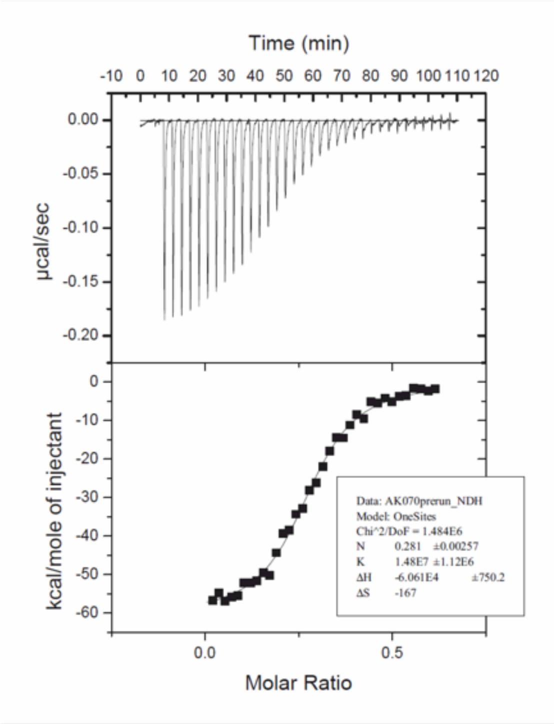

B3/pre-miR-21 measurement 3

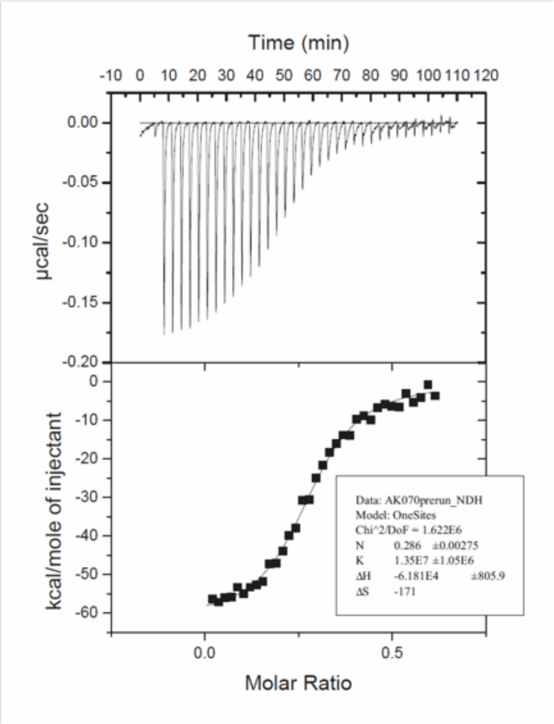

Figure S23: ITC measurements of *N*-terminally acetylated peptide **B3** with pre-miR-21-RNA. Measurements were performed in triplicate (*c*(peptide) = 4 µM and *c*(pre-miR-21-RNA) = 12 µM).

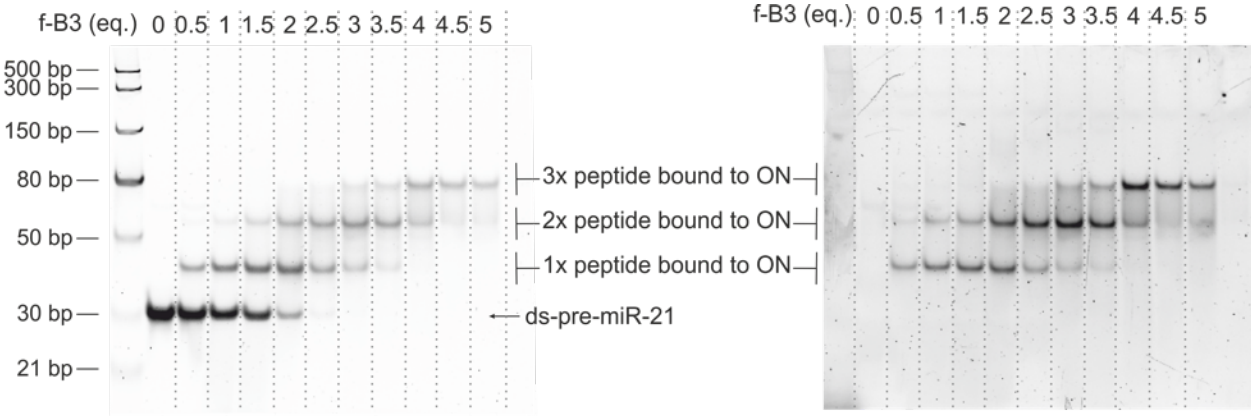

Figure S24: EMSA of pre-miR-21 (c = 1 µM) incubated with increasing concentrations of *N*-terminally fluorescein-labeled peptide **B3** (c = 0.5–5 µM). *Left,* gel imaged after SYBR^{TM} gold staining. *Right,* gel imaged for fluorescein fluorescence (ds = double-stranded, ON = pre-miR-21).

f-wt26

954.3 (found)
954.3 (calc. for $[M + 4H]^{4+}$)

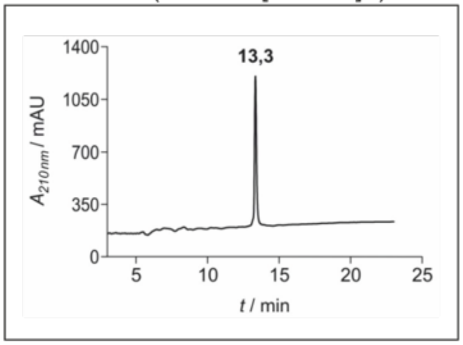

f-wt33

1171.5 (found)
1171.3 (calc. for $[M + 4H]^{4+}$

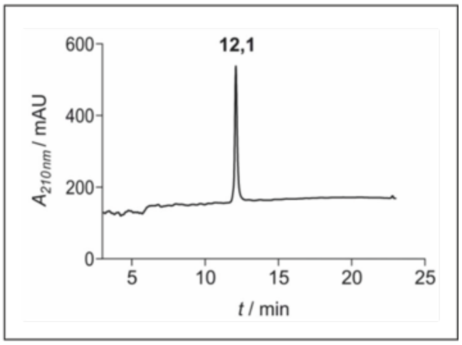

f-wt33Δ4

1033.8 (found)
1033.7 (calc. for $[M + 4H]^{4+}$)

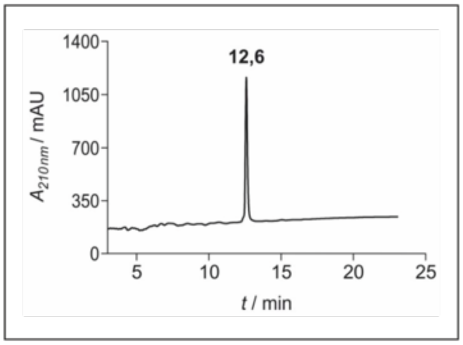

f-wt36

1260.2 (found)
1260.2 (calc. for $[M + 4H]^{4+}$)

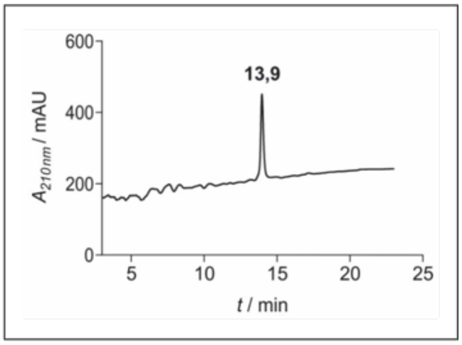

f-wt41

1431.0 (found)
1430.9 (calc. for $[M + 4H]^{4+}$)

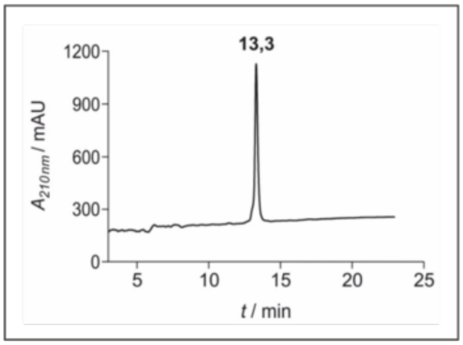

Figure S25: HPLC chromatograms (λ = 210 nm) including peak retention time and corresponding MS assignment of *N*-terminally fluorescein-labeled peptides (**f-wt26**, **f-wt33**, **f-wt33Δ4**, **f-wt36**, and **f-wt41**) used for initial EMSA truncation studies.

f-A1
1198.4 (found)
1198.4 (calc. for $[M + 4H]^{4+}$)

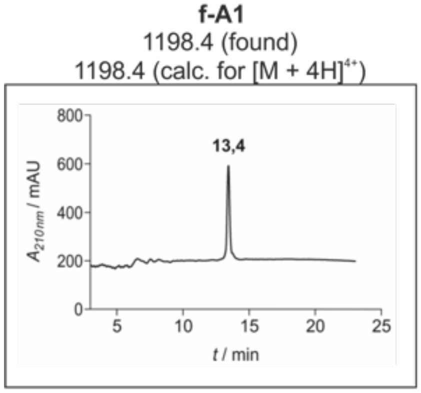

f-A2
1173.5 (found)
1173.6 (calc. for $[M + 4H]^{4+}$)

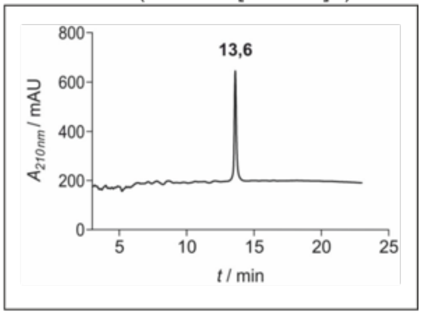

f-A3
1173.2 (found)
1173.4 (calc. for $[M + 4H]^{4+}$)

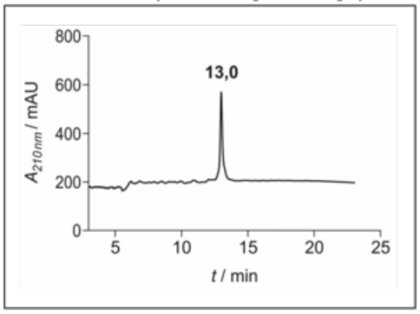

f-B
1169.8 (found)
1169.9 (calc. for $[M + 4H]^{4+}$)

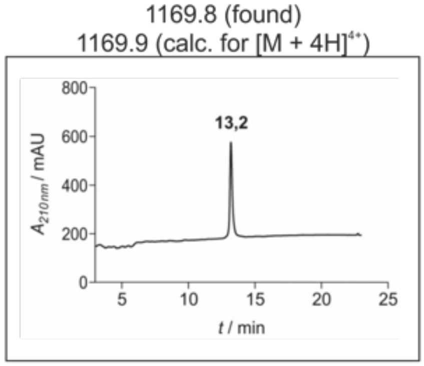

f-B1
1196.3 (found)
1196.7 (calc. for $[M + 4H]^{4+}$)

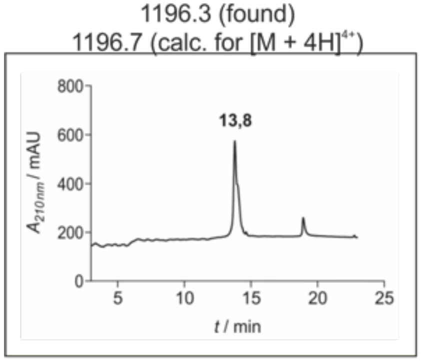

f-B2
1171.8 (found)
1172.1 (calc. for $[M + 4H]^{4+}$)

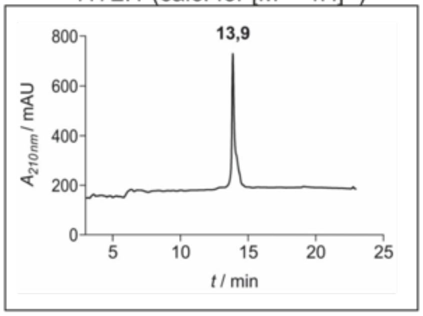

f-B3
1171.6 (found)
1171.9 (calc. for $[M + 4H]^{4+}$)

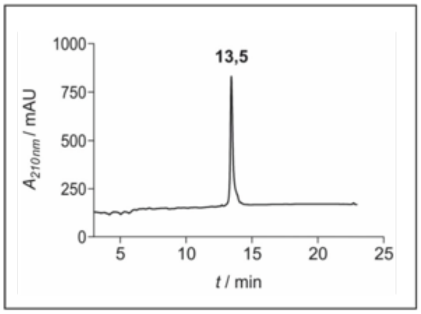

f-C
1183.9 (found)
1183.9 (calc. for $[M + 4H]^{4+}$)

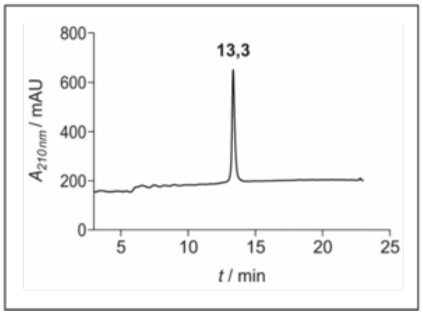

Figure S26: HPLC chromatograms (λ = 210 nm) including peak retention time and corresponding MS assignment of *N*-terminally fluorescein-labeled peptides (**f-A1**, **f-A2**, **f-A3**, **f-B**, **f-B1**, **f-B2**, **f-B3**, and **f-C**) used in stapled peptide EMSA screening, CD spectroscopy, protease stability assays, and flow cytometry.

f-C1

1210.9 (found)
1210.9 (calc. for $[M + 4H]^{4+}$)

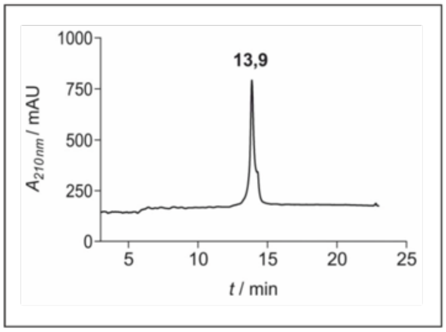

f-C2

1185.9 (found)
1186.1 (calc. for $[M + 4H]^{4+}$)

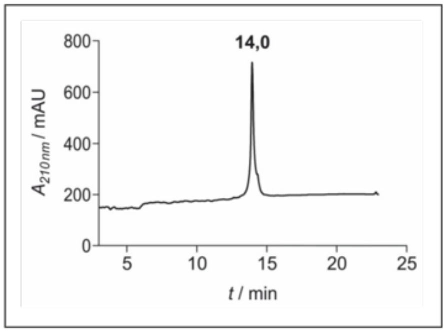

f-C3

1185.7 (found)
1185.9 (calc. for $[M + 4H]^{4+}$)

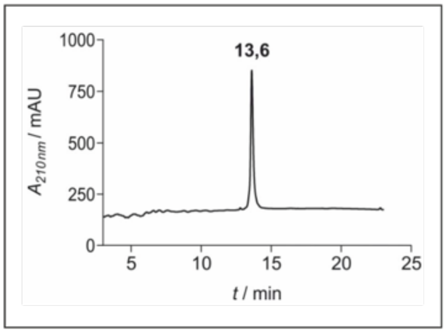

f-D1

1175.3 (found)
1175.1 (calc. for $[M + 4H]^{4+}$)

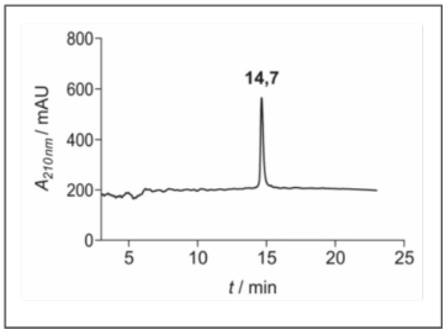

Figure S27: HPLC chromatograms (λ = 210 nm) including peak retention time and corresponding MS assignment of *N*-terminally fluorescein-labeled peptides (**f-C1**, **f-C2**, **f-C3**, and **f-D1**) used in stapled peptide EMSA screening, CD spectroscopy, protease stability assays, and flow cytometry.

wt33

1048.1 (found)
1048.2 (calc. for $[M + 4H]^{4+}$)

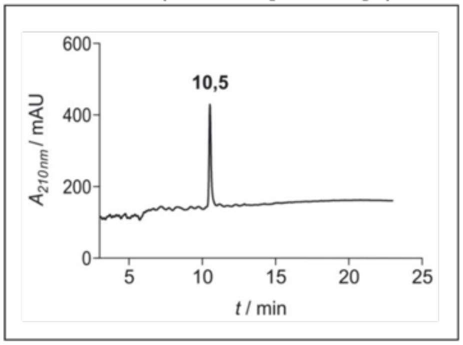

B1

1073.7 (found)
1073.5 (calc. for $[M + 4H]^{4+}$)

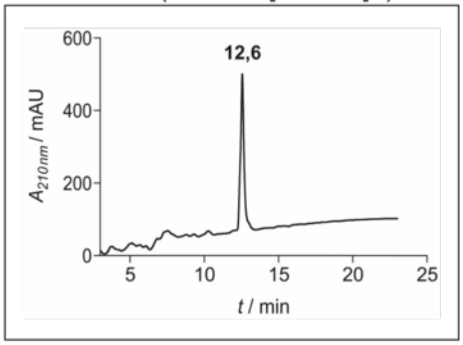

B3

1048.4 (found)
1048.5 (calc. for $[M + 4H]^{4+}$)

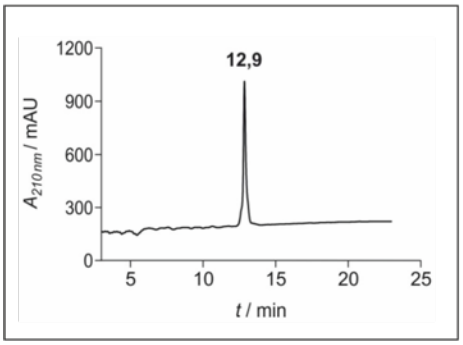

C1

1087.8 (found)
1087.8 (calc. for $[M + 4H]^{4+}$)

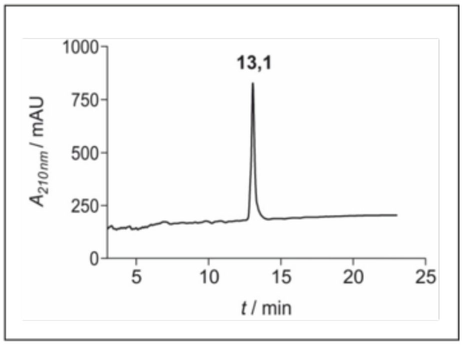

D1

1052.0 (found)
1052.0 (calc. for $[M + 4H]^{4+}$)

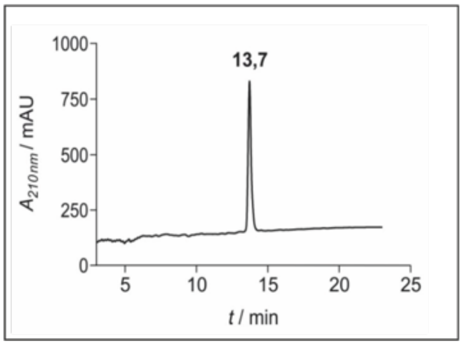

Figure S28: HPLC chromatograms (λ = 210 nm) including peak retention time and corresponding MS assignment of *N*-terminally acetylated peptides (**wt33**, **B1**, **B3**, **C1**, and **D1**) used in CD spectroscopy, ITC experiments and Dicer cleavage assays.

Bt-B3

1167.4 (found)
1167.4 (calc. for $[M + 4H]^{4+}$)

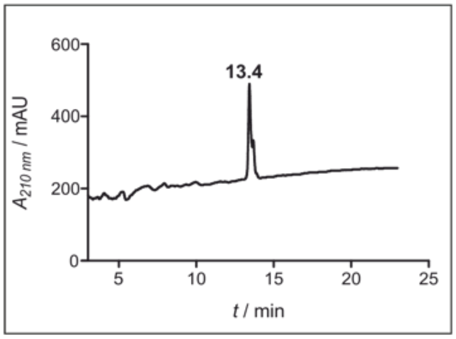

Bt-C1

1206.7 (found)
1206.7 (calc. for $[M + 4H]^{4+}$)

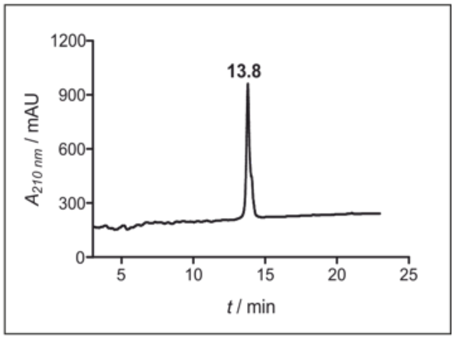

Bt-D1

1170.7 (found)
1170.9 (calc. for $[M + 4H]^{4+}$)

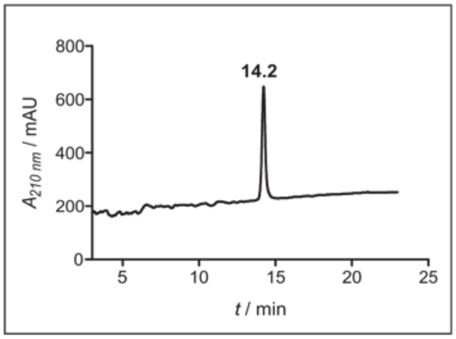

Figure S29: HPLC chromatograms (λ = 210 nm) including peak retention time and corresponding MS assignment of *N*-terminally biotinylated peptides (**Bt**-**B3**, **Bt**-**C1**, and **Bt**-**D1**) used in pull-down experiments.

B3a

1041.6 (found)
1041.7 (calc. for [M + 4H]$^{4+}$)

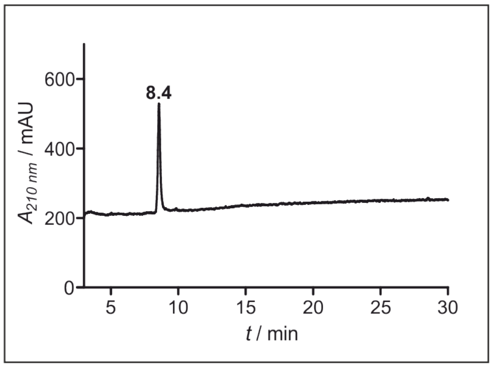

B3b

833.4 (found)
833.6 (calc. for [M + 5H]$^{5+}$)

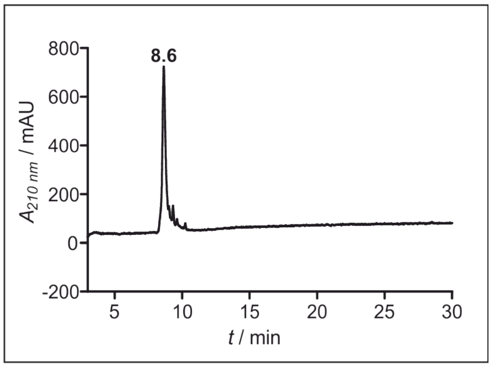

B3c

1034.6 (found)
1034.9 (calc. for [M + 4H]$^{4+}$)

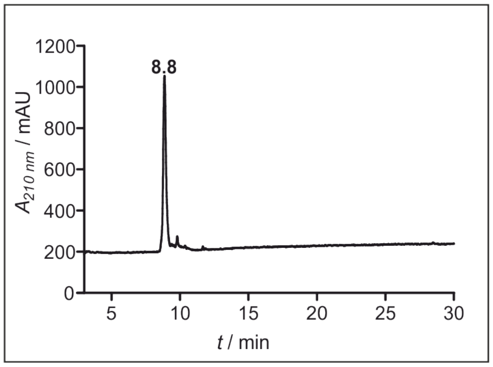

B3d

1034.7(found)
1034.9(calc. for [M + 4H]$^{4+}$)

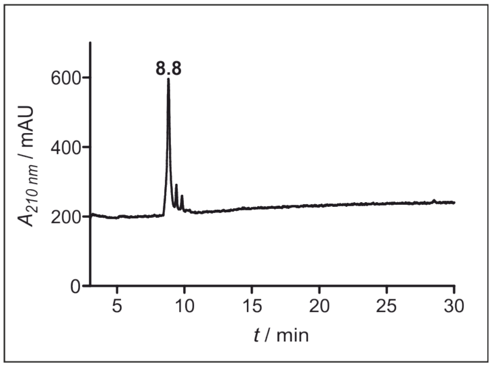

B3e

1027.9(found)
1028.2 (calc. for [M + 4H]$^{4+}$)

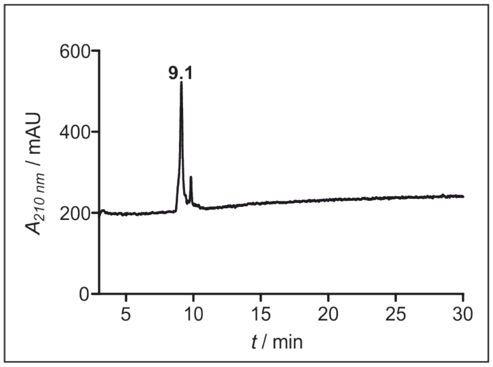

Figure S30: HPLC chromatograms (λ = 210 nm) including peak retention time and corresponding MS assignment of *N*-terminally acetylated **B3**-derived peptides (**B3a**, **B3b**, **B3c**, **B3d**, and **B3e**) used in EMSA experiments.

4. Supplementary References

1. Kim,Y.-W., Grossmann,T.N. and Verdine,G.L. (2011) Synthesis of all-hydrocarbon stapled α-helical peptides by ring-closing olefin metathesis. *Nat. Protoc.*, **6**, 761–771.

2. Poschner,B.C., Reed,J., Langosch,D. and Hofmann,M.W. (2007) An automated application for deconvolution of circular dichroism spectra of small peptides. *Anal. Biochem.*, **363**, 306–308.

3. Böhm,G., Muhr,R. and Jaenicke,R. (1992) Quantitative analysis of protein far UV circular dichroism spectra by neural networks. *Protein Eng. Des. Sel.*, **5**, 191–195.

4. Chen,H.-Y., Yang,J., Lin,C. and Yuan,Y.A. (2008) Structural basis for RNA-silencing suppression by Tomato aspermy virus protein 2b. *EMBO Rep.*, **9**, 754–60.
